# Crucial roles of the pentatricopeptide repeat protein SOAR1 in *Arabidopsis* response to drought, salt and cold stresses

**DOI:** 10.1007/s11103-015-0327-9

**Published:** 2015-06-21

**Authors:** Shang-Chuan Jiang, Chao Mei, Shan Liang, Yong-Tao Yu, Kai Lu, Zhen Wu, Xiao-Fang Wang, Da-Peng Zhang

**Affiliations:** MOE Systems Biology and Bioinformatics Laboratory, Center for Plant Biology, School of Life Sciences, Tsinghua University, Beijing, 100084 China

**Keywords:** Abscisic acid signaling, *Arabidopsis thaliana*, Pentatricopeptide repeat (PPR) protein, SOAR1, Salinity stress, Drought stress, Cold stress

## Abstract

Whereas several mitochondrial/chloroplast pentatricopeptide repeat (PPR) proteins have been reported to regulate plant responses to abiotic stresses, no nucleus-localized PPR protein has been found to play role in these processes. In the present experiment, we provide evidence that a cytosol-nucleus dual-localized PPR protein SOAR1, functioning to negatively regulate abscisic acid (ABA) signaling in seed germination and postgermination growth, is a crucial, positive regulator of plant response to abiotic stresses. Downregulation of *SOAR1* expression reduces, but upregulation of *SOAR1* expression enhances, ABA sensitivity in ABA-induced promotion of stomatal closure and inhibition of stomatal opening, and plant tolerance to multiple, major abiotic stresses including drought, high salinity and low temperature. Interestingly and importantly, the *SOAR1*-overexpression lines display strong abilities to tolerate drought, salt and cold stresses, with surprisingly high resistance to salt stress in germination and postgermination growth of seeds that are able to potentially germinate in seawater, while no negative effect on plant growth and development was observed. So, the *SOAR1* gene is likely useful for improvement of crops by transgenic manipulation to enhance crop productivity in stressful conditions. Further experimental data suggest that SOAR1 likely regulates plant stress responses at least partly by integrating ABA-dependent and independent signaling pathways, which is different from the ABI2/ABI1 type 2C protein phosphatase-mediated ABA signaling. These findings help to understand highly complicated stress and ABA signalling network.

**Electronic supplementary material:**

The online version of this article (doi:10.1007/s11103-015-0327-9) contains supplementary material, which is available to authorized users.

## Introduction

Terrestrial plant may suffer from various abiotic stresses during the whole life cycle, among which, salinity, drought and low temperature are major factors that restrict productivity. Plants have developed a series of resistance mechanisms to adverse environmental factors (Xiong and Zhu 2001; Zhu 2002, 2003). The phytohormone abscisic acid (ABA) plays central roles in modulating plant adaptation to various adverse conditions through regulating a set of stress response genes, which form a gene regulatory network to allow plants to cope with environmental stresses (Xiong and Zhu 2001; Zhu 2002; Finkelstein et al. 2002; Jakoby et al. 2002; Shinozaki et al. 2003; Adie et al. 2007; Cutler et al. 2010; Golldack et al. 2011; Fujita et al. 2011; Qin et al. 2011).

The pentatricopeptide repeat (PPR) superfamily proteins are encoded by one of the largest gene families in plants, which include about 450 members in *Arabidopsis thaliana* and more than 600 members in rice (*Oryza sativa*) (Small and Peeters 2000; Lurin et al. 2004; Rivals et al. 2006; Schmitz-Linneweber and Small 2008). PPR proteins are mostly targeted to mitochondria or chloroplasts, and involved in many aspects of RNA processing in these organelles, such as RNA splicing, editing, 5′/3′ end processing, stability, cleavage, and translation (Meierhoff et al. 2003; Williams and Barkan 2003; Lurin et al. 2004). The mitochondrial/chloroplast PPR proteins play diverse and important roles in plant developmental processes and responses to environmental stresses. An *Arabidopsis* chloroplast PPR protein SVR7 (Lv et al. 2014) was reported to be involved in photosynthesis and oxidative stress tolerance. Six *Arabidopsis* mitochondrial PPR proteins, PPR40 (Zsigmond et al. 2008), ABO5 (Liu et al. 2010), AHG11 (Murayama et al. 2012), SLG1 (Yuan and Liu 2012), PGN (Laluk et al. 2011), and SLO2 (Zhu et al. 2014), were reported to regulate ABA signaling and salt or drought stress responses. The *pgn* mutant and *PGN*-overexpression lines (Laluk et al. 2011), the *ppr40*-*1* (Zsigmond et al. 2008), *ahg11* (Murayama et al. 2012), *slg1* (Yuan and Liu 2012), and *slo2* (Zhu et al. 2014) mutants showed hypersensitivity to salt or osmotic stress during germination and/or postgermination growth, while adult plants of the *slo2* or *slg1* mutants showed increased drought and/or salt tolerance (Yuan and Liu 2012; Zhu et al. 2014). These data suggest a highly complicated mechanism by which these mitochondrial/chloroplast PPRs regulate plant response to abiotic stresses, though it was proposed that they may regulate reactive oxygen species (ROS) homeostasis to be involved in stress responses or ABA signaling.

Whereas numerous PPR proteins are found to be localized to the mitochondrial or chloroplast intracellular space, few PPR proteins have been found to reside in other cellular compartments such as cytosol or nucleus. To our knowledge, so far, only two PPR proteins were found in the nucleus, which regulate embryogenesis (Ding et al. 2006; Hammani et al. 2011). Up to date, however, no nucleus- or cytosol-localized PPR protein has been found to regulate plant responses to abiotic stresses. Most recently, we identified a cytosol-nucleus dual-localized PPR protein, SOAR1 (for suppressor of the *ABAR*-overexpressor 1), which functions negatively in ABA signaling in seed germination and seedling growth downstream of the Mg-chelatase H subunit/putative ABA receptor (CHLH/ABAR) and upstream of an important ABA-responsive bZIP transcription factor ABI5 (Jiang et al. 2014; Mei et al. 2014; Wang and Zhang 2014). However, it remains still unknown whether and how SOAR1 regulates plant response to abiotic stresses. In the present experiment, we show that SOAR1 is a crucial, positive regulator of plant response to multiple, major abiotic stresses including drought, high salinity and low temperature. The *SOAR1*-overexpression lines display strong abilities to tolerate drought, salt and cold stresses, which is likely useful for improvement of crops. Further experimental data suggest that SOAR1 likely regulates plant stress responses at least partly by integrating ABA-dependent and independent signaling pathways. These findings help to understand highly complicated stress and ABA signalling network.

## Materials and methods

### Plant materials and growth conditions

The T-DNA insertion mutants *soar1*-*2* (stock no. FLAG_546D07) and *soar1*-*3* (stock no. FLAG_500B04) in *SOAR1* gene (At5g11310, see Mei et al. 2014) were obtained from Versailles Genetics and Plant Breeding Laboratory, *Arabidopsis thaliana* Resource Centre (INRA; http://dbsgap.versailles.inra.fr/portail/) with the Col ecotype as background. The *abi1*-*3 abi2*-*2* double mutant seeds are a generous gift from Dr. Y. Guo (China Agricultural University, Beijing, China), where *abi1*-*3* (stock no. SALK_076309) and *abi2*-*2* (stock no. SALK_015166) mutants, two T-DNA insertion knockout mutants in the *ABI1* (At4g26080) and *ABI2* (At5g57050) genes, respectively, were obtained from the Arabidopsis Biological Resource Centre (ABRC) with the Col ecotype as background. These mutants were identified previously as we described (Mei et al. 2014).

The *Arabidopsis* ecotype Col-0 was used to generate transgenic plants. The *SOAR1*-overexpression lines and *ABI2*-overexpression line ABI2-OE were generated as described previously (Sun et al. 2011; Mei et al. 2014). Plants were grown on Murashige and Skoog (MS) medium (Murashige and Skoog 1962; PhytoTechnology, Shawnee Mission, KS, USA) containing 3 % (w/v) sucrose and 0.8 % (w/v) agar or in compost soil under a 16 h photoperiod in the growth chamber or phytotron at about 20 °C.

### Seed germination and postgermination growth

Seeds were surface sterilized in 4 % (v/v) sodium hypochlorite, and rinsed five times with sterile water. The seeds were sown on MS basal medium as mentioned above, with addition of NaCl or D-mannitol (Amresco, Solon, OH, USA) at indicated concentrations for assaying salt and osmotic stress responses. The seeds were stratified at 4 °C for 3 day, and transferred to 20 °C under long-day cycle (16 h/8 h light/dark) for phenotypic analysis of germination and post-germination growth.

### Stomatal movement

Mature rosette leaves from about 4-week-old plants were used for assays of stomatal movement. The stomatal apertures on the abaxial surface of leaves were measured. To observe ABA-induced stomatal closure, leaves were immersed in a buffer consisting of 50 mM KCl and 10 mM MES-KOH with pH 6.15, and then exposed to cold light source (Chongqing Optec Instrument Co., Lt, Chongqing, China) for 2.5 h. Subsequently, the leaves were transferred into the fresh buffer as described above but supplemented with 0 (control) or 20 μM (±) ABA (Sigma, Saint Louis, MO, USA) for an incubation of 2.5 h before stomatal apertures were measured. To study ABA-inhibited stomatal opening, leaves were kept in the dark for 2.5 h, and then exposed to light and incubated for 2.5 h in the buffer as mentioned above and supplemented with 0 (control) or 20 μM (±) ABA before stomatal apertures were measured. The assays were performed with three independent repetitions (*n* ≥ 80 apertures per experiment).

### Water loss from detached leaves

Mature rosette leaves of similar size were sampled from about 4-week-old plants and placed in culture dishes under light at room temperature (about 24 °C) with the relative humidity of air about 40 %. Water loss was evaluated by weighing leaves at the indicated time points. The assays were performed with three independent repetitions.

### Drought treatment

Ten-d-old seedlings were transplanted to 7-cm pots filled with compost soil with the same water content. Plants were grown at 22 °C under long-day cycle (light/dark: 16 h/8 h) for 1 week, and drought was imposed on the plants by withholding water for about 3 weeks until the lethal effect was observed on the mutant plants, while the control plants were well watered. The growth status and survival rates of different genotypes were recorded 3 days after the plants were re-watered. The entire experiment was replicated three times with similar results.

### Salt treatment

To investigate germination and postgermination growth of different genotypes under salt stress, seeds were sown directly on MS medium containing 130, 150, 175, or 200 mM NaCl and grown 18 days after stratification, and then root length and fresh weight of seedlings were recorded. To test the extremity of the NaCl concentrations which the *SOAR1*-overexpression lines tolerate, seeds were sown in the MS medium containing 250, 300, 350, 422, and 513 mM NaCl, and germination and postgermination growth were investigated 2 weeks after stratification. To assay of salt response of the whole mature plants, NaCl treatment was imposed with irrigation as described previously (Shi et al. 2003) with some modifications. Ten-day-old seedlings were transferred to soil and continued to grow 1 week before the NaCl treatment. NaCl solution was imposed with three increasing concentrations (100, 150, and 200). With each NaCl concentration the plants were irrigated one time at an interval of 4 days, and for a total period of 12 days plants were irrigated three times with corresponding concentrations of NaCl, and then they continued to be irrigated with 200 mM NaCl solution tow times at an interval of 4 days, and were investigated for salt tolerance 7 days after the last NaCl irrigation. The control plants were irrigated with water.

### Freezing treatment

The freezing treatment was performed as described previously (Shi et al. 2012) with some modifications. Two-week-old seedlings grown in petri dishes at 20 °C were used for the cold-acclimation (CA) treatment and non-acclimated (NA) treatment. Seedlings of the NA group were subjected directly to a freezing treatment, while seedlings of the CA group were acclimated at 4 °C for 7 days before the freezing treatment. Seedlings were placed in a freezing chamber (RuMED4001, Stuttgart, Germany) set at 0 °C programmed to cool at 1 °C per hour until the minimum temperature. Petri dishes of plants were removed at the indicated time or temperature points. After the freezing treatment, the plants were incubated at 4 °C in the dark for 12 h and then transferred to light at 20 °C in the growth chamber. The survival rates of the seedlings were scored visually after recovering for about 2 days. In addition, the electrolyte leakage and proline content of the plants treated by freezing were measured according to the procedures described previously (Bates et al. 1973; Lee et al. 2001; Yang et al. 2010).

### Quantitative real-time PCR

Quantitative real-time PCR for the osmosis-, salt- and cold-responsive genes (see Supplementary Table S1 online for the gene-specific primers) was performed essentially as described previously (Shang et al. 2010; Mei et al. 2014), with the Bio-Rad Real-Time System CFX96TM C1000 thermal cycler (Bio-Rad, Hercules, CA, USA) and following the manufacture’s instructions. Total RNA was isolated with plant total RNA extraction kit (BioTeke Corporation, Beijing, China) supplemented with DNA digestion (New England Biolabs RNase-Free DNase I, Beijing, China), and then the RNA sample was reverse-transcribed with the Transcriptor First Strand cDNA Synthesis Kit (Roche, Mannheim, Germany) according to the manufacturer’s instructions. Amplification of *ACTIN2/8* genes was used as an internal control. The cDNA was amplified using SYBR Premix Ex Taq (TaKaRa, Dalian, China) with the CFX96TM C1000 thermal cycler in a 10 ml volume. The relative expression levels were calculated as described (Mei et al. 2014). It is noteworthy that we used two *soar1* mutant and two *SOAR1*-overexpresssion lines to carry this experiment, and got the similar results. We presented the data of one mutant and one OE line as a representative.

## Results

### Downregulation of *SOAR1* expression reduces, but upregulation of *SOAR1* expression enhances, dehydration tolerance

**Fig. 1:**
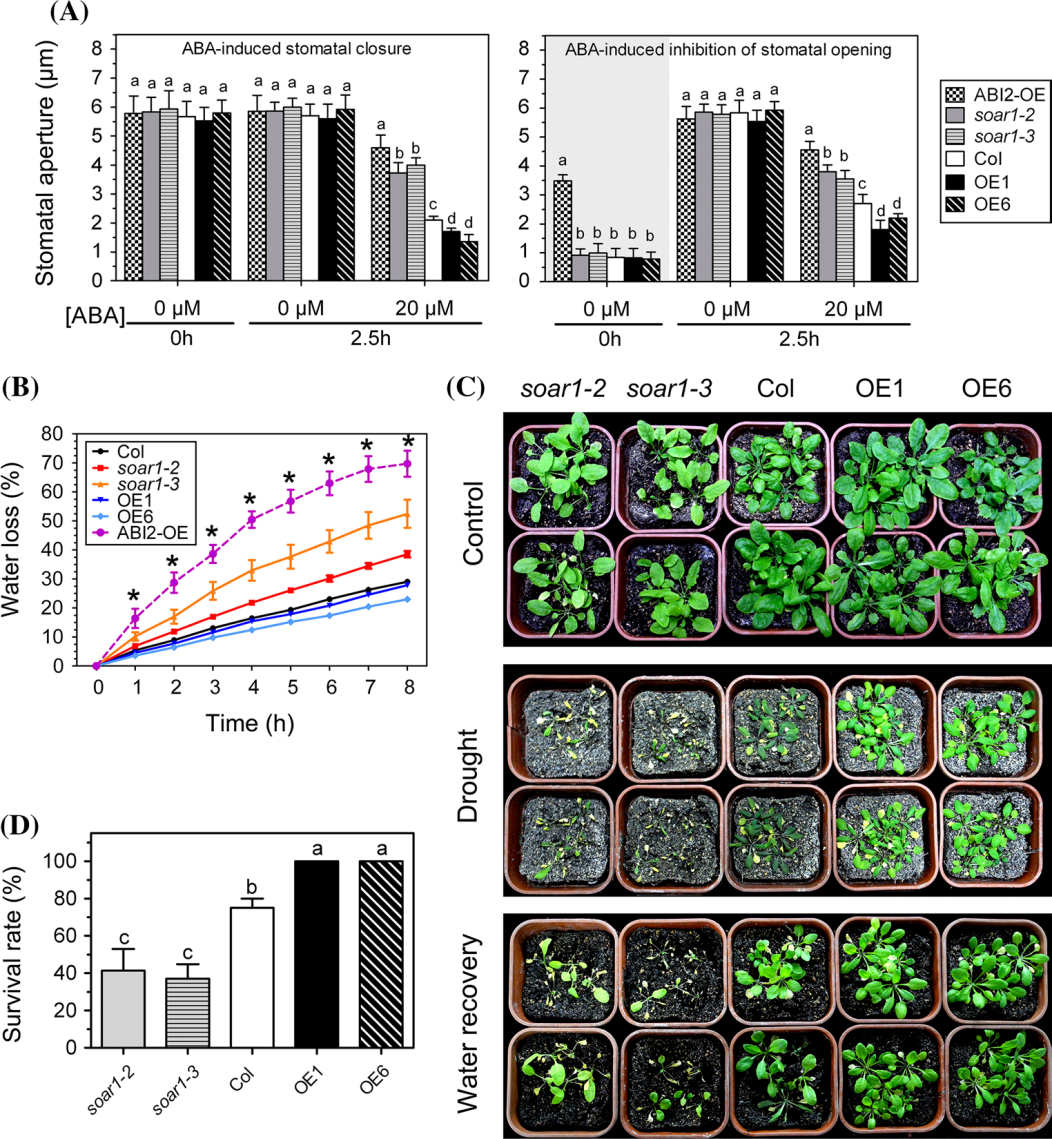
SOAR1 positively regulates plant resistance to drought stress. **a** ABA-induced stomatal closure (*left panel*) and inhibition of stomatal opening (*right panel*) of the wild-type Col, two *SOAR1*-knockdown mutant alleles *soar1*-*2* and *soar1*-*3*, *SOAR1*-overexpressing lines OE1 and OE6, and an *ABI2*-overexpressing line ABI2-OE. Mature rosette leaves from 4-week-old seedlings were used for the assays. Values are the mean ± SE from three independent experiments (n ≥ 80 apertures per experiment), and different letters indicate significant differences at *P* < 0.05 (Duncan’s multiple range test) when comparing values within the same ABA concentration. **b** Water loss rates during a 6-h period from the detached leaves of the different genotypes described in (**a**). Values are the mean ± SE of five independent experiments. Star indicates that significant differences at *P* < 0.05 (Duncan’s multiple range test) exist when comparing values within the same time point. The entire experiment was replicated five times with similar results. **c** Plant growth status in the drought assays. Drought was imposed on the wild-type Col, *soar1*-*2* and *soar1*-*3* mutants, as well as OE1 and OE6, by withholding water for about 3 weeks until the lethal effect was observed on the mutant plants, while the control plants were well watered. The growth status was recorded 3 days after the plants were re-watered. The entire experiment was replicated three times with similar results. **d** Survival rate of different genotypes as mentioned in (**c**). Drought was imposed on the plants by withholding water until the lethal effect was observed on the mutant plants, then survival rate was recorded 3 days after the plants were re-watered. Values are the mean ± SE from three independent experiments (n ≥ 50 plants per line for each experiment) and different letters indicate significant differences at *P* < 0.05 (Duncan’s multiple range test)

ABI2 is a member of the clade-A type-2C protein phosphatases (PP2Cs) that regulates negatively ABA signaling and stress responses (Leung et al. 1997; Schweighofer et al. 2004; Cutler et al. 2010; Liang and Zhang 2014). We observed that the stomata of an *ABI2*-overexpression line ABI2-OE that we generated previously (Sun et al. 2011; Mei et al. 2014) kept open even in the dark and exhibited strong ABA-insensitive phenotypes in ABA-induced promotion of stomatal closure and inhibition of stomatal opening (Fig. 1a), revealing that guard cell signaling in response to ABA was seriously lesioned in the ABI2-OE line. Similar to this *ABI2*-overexpression line, the two *SOAR1*-knockdown mutant alleles *soar1*-*2* and *soar1*-*3* showed ABA-insensitive phenotypes in ABA-induced promotion of stomatal closure and inhibition of stomatal opening, of which the intensity was slightly weaker than that of the ABI2-OE line (Fig. 1a). In contrast to the *soar1*-*2* and *soar1*-*3* mutants, the *SOAR1*-overexpression lines OE1 and OE6 showed ABA-hypersensitive phenotypes in ABA-induced promotion of stomatal closure and inhibition of stomatal opening (Fig. 1a). These data show that SOAR1 positively regulates guard cell signaling in response to ABA.

Essentially consistent with the ABA-related phenotypes in stomatal movement, the detached leaves of *soar1*-*2* and *soar1*-*3* mutants, as well as those of the ABI2-OE line, showed higher rates, but the *SOAR1*-overexpression line OE6 showed lower rates, of water loss than those of the wild-type plants under dehydration conditions (Fig. 1b, Supplementary Table S2). It was noted, however, that the *SOAR1*-overexpression line OE1 showed no significant difference from the wild-type plants in relation to the water loss from detached leaves (Fig. 1b, Supplementary Table S2). Interestingly and importantly, the *soar1*-*2* and *soar1*-*3* mutants showed significantly more sensitive to drought than the wild-type plants, while both *SOAR1*-overexpression lines showed higher capacity to conserve water and to tolerate drought stress condition (Fig. 1c, d). It is noteworthy, however, that the amplitude of change in water loss from detached leaves of the *SOAR1*-overexpression lines was relatively small compared with wild types, while this change resulted in significant increase in their drought tolerance. We did not observe differences in stomata number between wild-type plants and *SOAR1*-overexpression lines. So, overexpression of SOAR1 may induce additional mechanism, such as enhanced ability of cells to tolerate osmotic stress, to allow plants to cope with drought environment.

### Downregulation of *SOAR1* expression reduces, but upregulation of *SOAR1* expression enhances, osmotic and salt tolerance in seed germination and postgermination growth

**Fig. 2:**
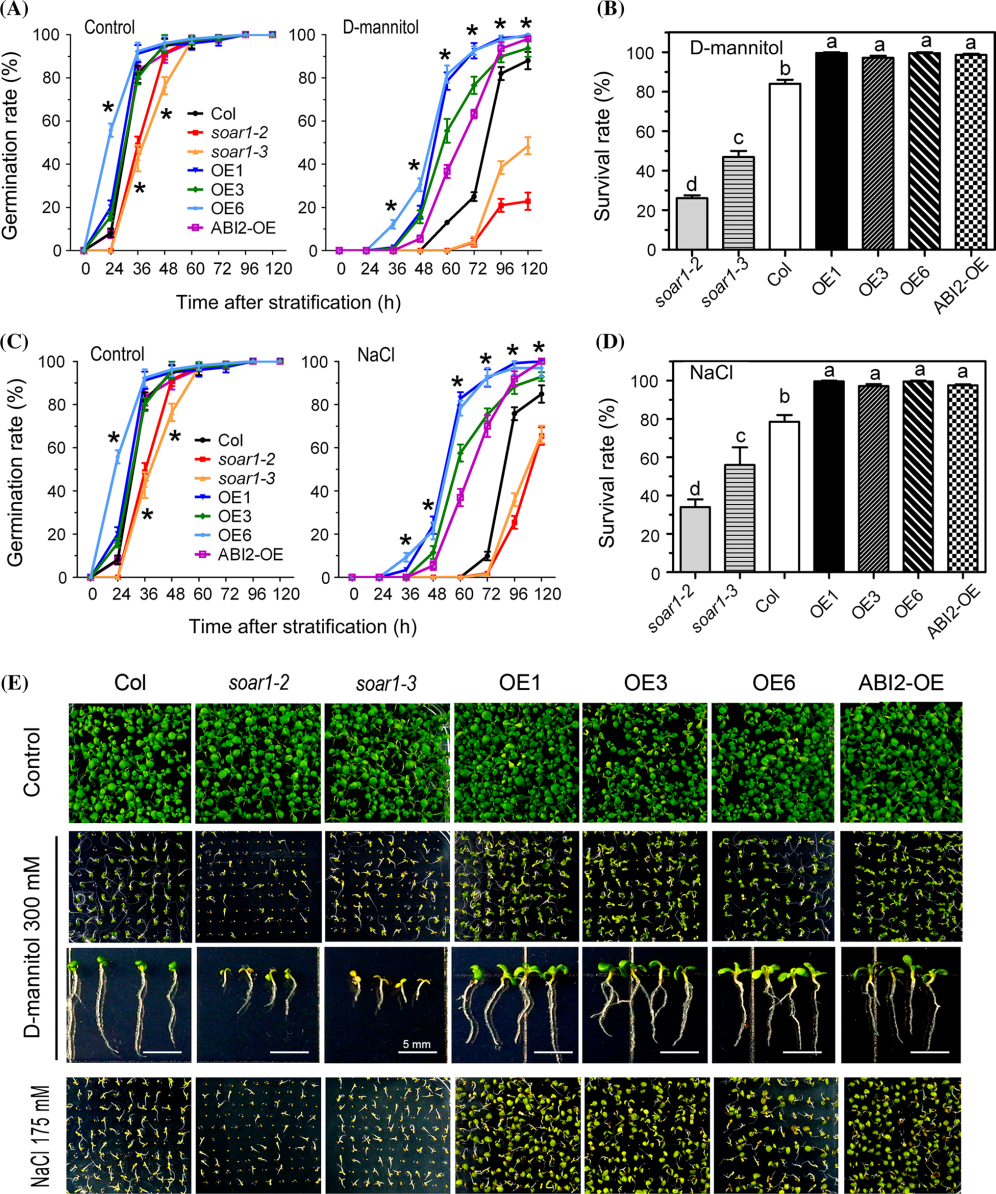
Seed germination and postgermination growth of different genotypes under D-mannitol-induced osmotic stress and salt stress. **a** Seed germination rates of the wild-type Col, two *SOAR1*-knockdown mutant alleles *soar1*-*2* and *soar1*-*3*, three *SOAR1*-overexpression lines (OE1, OE3 and OE6), and the *ABI2*-overexpression line (ABI2-OE). Seeds were sown in the mannitol-free (control) and D-mannitol-containing (300 mM) MS-medium, and the germination rates were recorded from 24 to 120 h after stratification at 4 °C for 3 days. **b** Survival rate of different genotypes as mentioned in (A) grown on the mannitol-containing medium 7 days after stratification. **c** Seed germination rates of the genotypes as described in A. Seeds were sown in the NaCl-free (control) and NaCl-containing (175 mM) MS-medium, and the germination rates were recorded from 24 to 120 h after stratification at 4 °C for 3 days. **d** Survival rate of different genotypes as mentioned in (**a**) grown on the NaCl-containing medium (175 mM) 7 days after stratification. **e** Postgermination growth of the different genotypes described in (**a**) in the mannitol/NaCl-free (control) and 300 mM-D-mannitol- or 175 mM-NaCl-containing medium 7 days after stratification. Bars indicate 5 mm. Each value in A-D is the mean ± SE of three independent experiments, and stars in A and C indicate that significant differences at *P* < 0.05 (Duncan’s multiple range test) exist when comparing values within the same time point, while different letters in B and D indicate significant differences at *P* < 0.05 (Duncan’s multiple range test) between different genotypes

We investigated phenotypes of the *soar1* mutants and the *SOAR1*-overexpression lines subjected to both the osmotic stress induced by application of 300-mM mannitol in the medium and salt stress in the 175-mM NaCl-containing medium where the seeds of the different genotypes were directly sown. We observed that, compared with the wild-type Col seeds, the germination rates of seeds, survival rates of the germinating seeds, and the subsequent early-postgermination growth (7 d after stratification) of *soar1*-*2* and *soar1*-*3* decreased, but those of the *SOAR1*-overexpression lines OE1, OE3 and OE6 increased under both stressful conditions (Fig. 2a–e, Supplementary Tables S3-S5). The *ABI2*-overexpressing line ABI2-OE showed similar mannitol/NaCl-insensitive phenotypes to those of the *SOAR1*-overexpression lines (Fig. 2a–e, Supplementary Tables S3-S5).

**Fig. 3:**
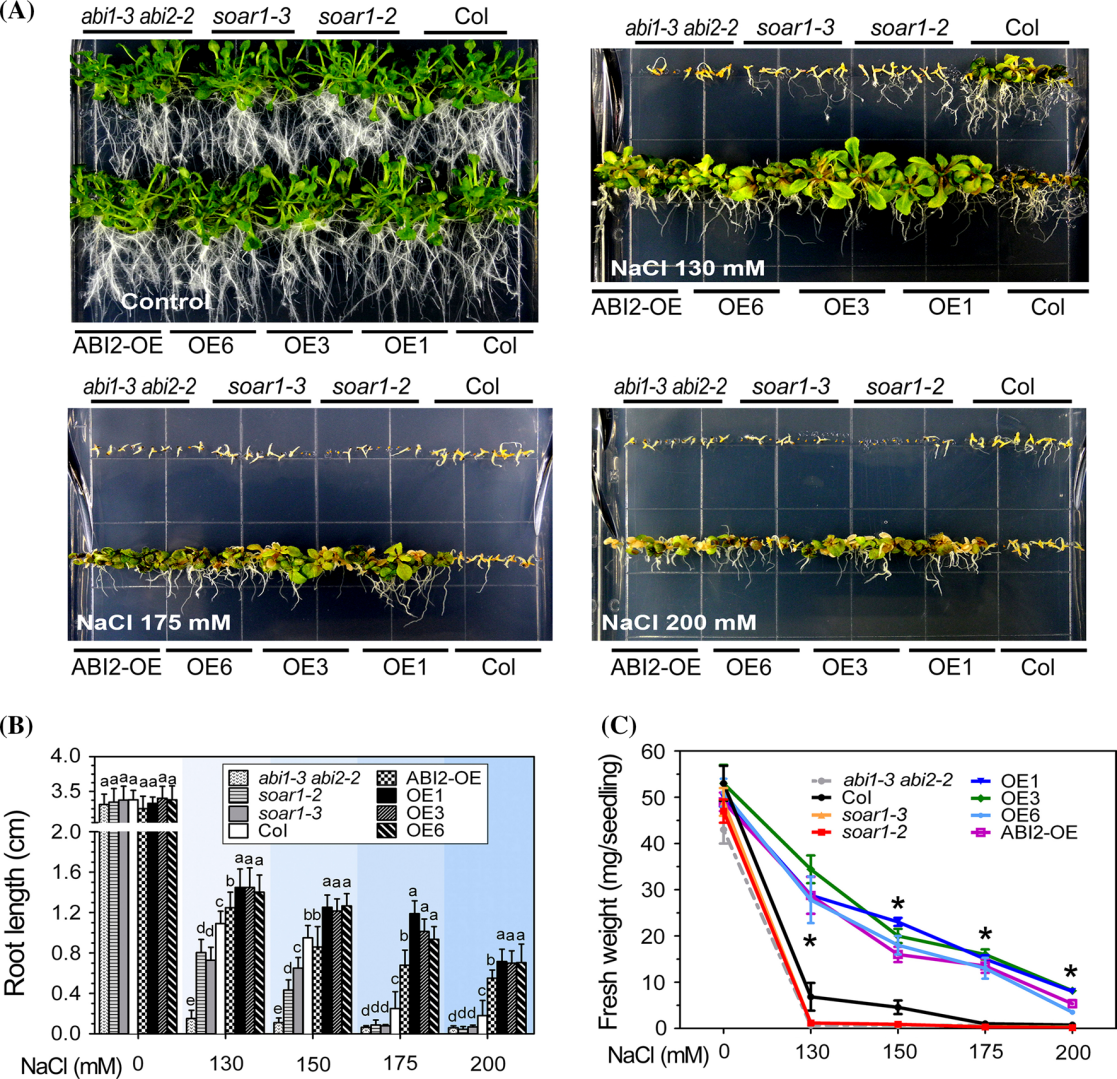
Postgermination growth of different genotypes under salt stress. **a** Postgermination growth of the wild-type Col, two *SOAR1*-knockdown mutant alleles *soar1*-*2* and *soar1*-*3*, three *SOAR1*-overexpression lines (OE1, OE3 and OE6), *abi1*-*3 abi2*-*2* double knockout-mutant, and the *ABI2*-overexpression line (ABI2-OE) in the MS medium containing 0 (control), 130, 175, and 200 mM NaCl (18 days after stratification). The entire experiment was replicated three times with similar results. **b**, **c** Statistical values of the root length (**b**) and fresh weight (**c**) of the different genotypes described in (A) grown in the medium supplemented with 0, 130, 150, 175, and 200 mM NaCl. Each value is the mean ± SE of five biological determinations with different letters (**b**) or stars (**c**) indicating significant differences at *P* < 0.05 (Duncan’s multiple range test) when comparing values within the same NaCl concentration

Further, NaCl was applied at different concentrations in the medium where the seeds were directly sown to test the responses of these genotypes to salt stress in postgermination growth during a relatively prolonged period (18 d after stratification). We observed that, in the medium containing 130, 175 or 200 mM NaCl, the postgermination growth of the *soar1*-*2* and *soar1*-*3* mutants and *abi1*-*3 abi2*-*2* double mutant (knockout mutant of *ABI1* and *ABI2* genes) was significantly reduced, while that of the *SOAR1*-overexpression lines OE1, OE3 and OE6 as well as the ABI2-OE line was significantly enhanced in comparison of wild-type seedlings (Fig. 3a–c, Supplementary Fig. S1, Supplementary Table S6). These data are essentially consistent with the above-mentioned observations (Fig. 2).

### *SOAR1*-overexpression results in resistance of seed germination to extremely high salinity, and in salt insensitivity of mature plants, in contrast to salt hypersensitivity resulting from *SOAR1* down-expression

**Fig. 4:**
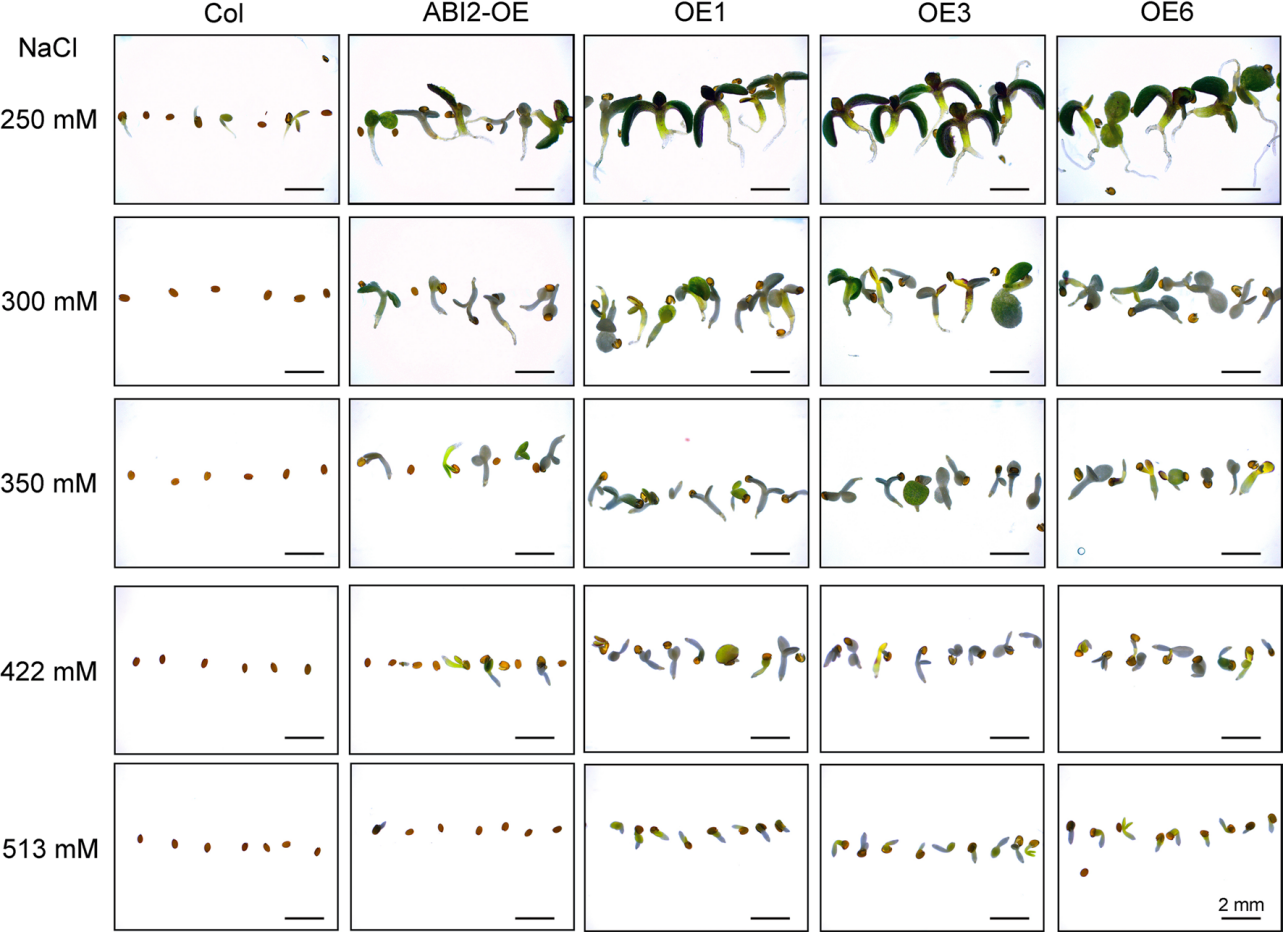
Test of the extremity of the NaCl concentrations under which seeds of the *SOAR1*-overexpression lines germinate and continue to grow. Germination and postgermination growth of the wild-type Col, the *ABI2*-overexpression line (ABI2-OE) and three *SOAR1*-overexpression lines OE1, OE3 and OE6 in the MS medium containing 250, 300, 350, 422, and 513 mM NaCl, were investigated 2 weeks after stratification. The entire experiment was replicated three times with similar results. Bars indicate 2 mm

We tested the extremity of NaCl concentrations under which the *SOAR1*-expression lines germinate and continue to grow by applying NaCl at different concentrations (250, 300, 350, 422, and 513 mM) in the medium in which the seeds were directly sown. Surprisingly, we observed that the seeds of the *SOAR1*-expression lines OE1, OE3 and OE6 germinated even in the medium containing higher than 500 mM of NaCl, and continued postgermination growth in the medium containing higher than 350 mM of NaCl, whereas the wild-type seeds scarcely germinated at the medium containing 250 mM of NaCl (Fig. 4, Supplementary Fig. S2). The phenotypes of the *ABI2*-overexpressing line ABI2-OE showed similar to, but weaker than, those of the *SOAR1*-overexpression lines (Fig. 4, Supplementary Fig. S2). The NaCl concentrations of 422 mM and 513 mM approximate those of artificial seawater (Lyman and Fleming 1940; Kester et al. 1967; Veerman et al. 2009), suggesting that the seeds of the *SOAR1*-expression lines may germinate in seawater.

**Fig. 5:**
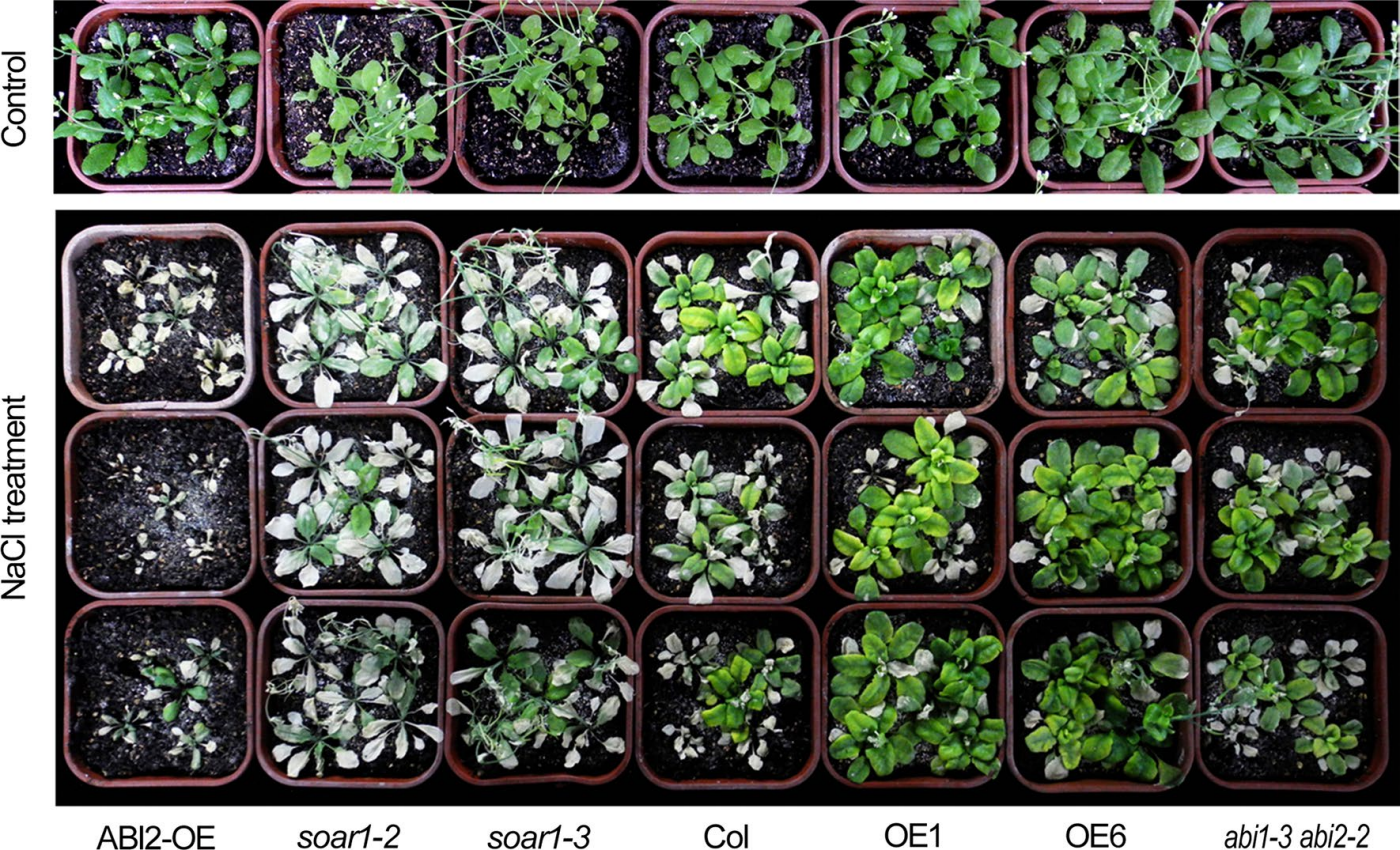
Growth status of the whole mature plants of different genotypes under salt stress. Growth of wild-type Col, *soar1*-*2* and *soar1*-*3* mutants, *abi1*-*3 abi2*-*2* double knockout-mutant, the *ABI2*-overexpression line ABI2-OE, and *SOAR1*-overexpression lines OE1 and OE6 is shown after NaCl treatment. Ten-day-old seedlings were transferred to soil and continued to grow 1 week before the NaCl treatment. NaCl solution was imposed with three increasing concentrations (100, 150, and 200). With each NaCl concentration the plants were irrigated one time at an interval of 4 days, and for a total period of 12 days plants were irrigated three times with corresponding concentrations of NaCl, and then they continued to be irrigated with 200 mM NaCl solution two times at an interval of 4 days, and the pictures were taken 7 days after the last NaCl irrigation. The control plants were irrigated with water. The entire experiment was replicated three times with similar results

Interestingly, further experiments of salt stress, imposed on the mature, whole plants by irrigation with NaCl solution, showed that the mature plants of the *SOAR1*-expression lines OE1, OE3 and OE6 were insensitive, but those of the *soar1*-*2* and *soar1*-*3* mutants were hypersensitive, to salt stress in comparison with wild-type plants (Fig. 5). It is particularly noteworthy that, in contrast to the mannitol/NaCl-insensitive phenotypes of their seeds sown directly in the mannitol or NaCl-containing medium, the mature plants of the *ABI2*-overexpressing line ABI2-OE showed highly sensitive to NaCl (Fig. 5). However, the mature plants of the *abi1*-*3 abi2*-*2* double mutant showed NaCl-insensitive phenotype, which is similar to, but weaker than, that of the *SOAR1*-expression lines (Fig. 5). These data suggest that SOAR1 regulates plant response against abiotic stress by mechanisms at least partly different from those used by PP2Cs like ABI2.

### Downregulation of *SOAR1* expression reduces, but upregulation of *SOAR1* expression enhances, freezing tolerance

**Fig. 6:**
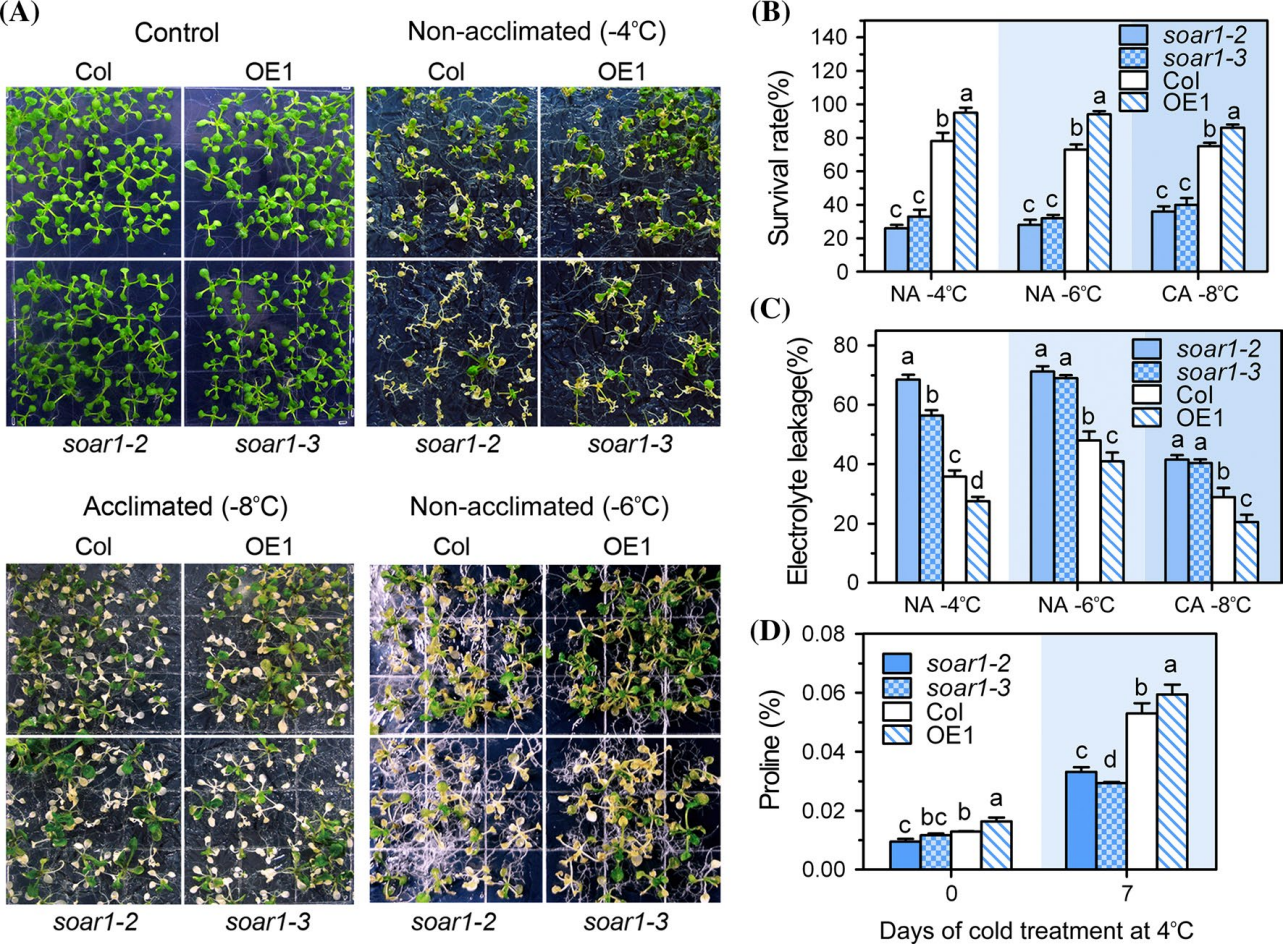
Changes in growth, electrolyte leakage and proline contents of different genotypes under freezing condition. **a, b** Freezing assay of wild-type Col, *soar1*-*2*, *soar1*-*3*, and *SOAR1*-overexpression line (OE1). Two-week-old seedlings were placed directly under freezing condition (non-acclimated plants) at −4 °C (*top*, right in A, and NA −4 °C in B) or −6 °C (*bottom*, right in A, and NA −6 °C in B) for 1 h, while other two-week-old seedlings were firstly acclimated at 4 °C for 7 days and then placed under a freezing condition at −8 °C for 1 h (cold-acclimated plants, bottom, left in A, and CA −8 °C in B). The growth and survival rates of different genotypes were recorded after a 2-days recovery at 20 °C and are shown in A and B, respectively. The control plants were grown at 20 °C. Each value in B is the mean ± SE of three biological determinations and different letters indicate significant differences at *P* < 0.05 (Duncan’s multiple range test) within the same treatment. **c** Ion leakage of different genotypes described in A and B after freezing treatment. Each value is the mean ± SE of three independent determinations and different letters indicate significant differences at *P* < 0.05 (Duncan’s multiple range test) within the same treatment. **(D)** Proline content in different genotypes described in A. The plants were grown at 20 °C for 2 weeks, acclimated at 4 °C for 7 days, and sampled for analysis. Each value is the mean ± SE of three independent determinations and different letters indicate significant differences at *P* < 0.05 (Duncan’s multiple range test) within the same time point

For the freezing assays, 2-week-old seedlings of the non-cold-acclimated group were subjected directly to a progressive freezing process, but those of the cold-acclimated group were treated with the progressive freezing process after a pretreatment of so-called cold-acclimation (see Materials and methods), and then growth status and survival rates were recorded to estimate the freezing consequences. We observed that, in comparison with wild-type plants, the *soar1*-*2* and *soar1*-*3* mutants showed freezing-sensitive phenotypes, while the *SOAR1*-overexpression line OE1 showed freezing-tolerant phenotypes, as evidenced by the data of growth status (Fig. 6a) and especially survival rates after freezing (Fig. 6b), which is true regardless of the cold- or non-cold- acclimation pretreatment (Fig. 6a, b), suggesting that SOAR1 positively regulates both the basal and acquired freezing tolerance. Consistent with these observations, the *soar1*-*2* and *soar1*-*3* mutants showed higher electrolyte leakage and lower level of proline, and the *SOAR1*-overexpression line showed lower electrolyte leakage and higher level of proline particularly following the freezing treatments (Fig. 6c, d), indicating that the cell membranes of the *soar1*-*2* and *soar1*-*3*mutants may be damaged by the freezing treatment, and the cells of the *SOAR1*-overexpression line may be protected from freezing partly by a lower level of electrolyte leakage from cells and a higher level of proline in cells.

### Changes in *SOAR1* expression alter expression of a subset of genes involved in osmotic, salt and cold responses

We tested *SOAR1* expression in wild-type plants under salt and cold stresses before assessing a series of known stress-responsive genes, and found that the *SOAR1* expression levels increased with salt stress, which was similar to those of *SOS1* and *SOS2*, two well-characterized genes positively involved in plant response to salt (Liu et al. 2000; Qiu et al. 2002; Shi et al. 2003) (Supplementary Fig. S3). Cold treatment at 4 °C induced a transient, but that at 0 °C induced a constant, increase of *SOAR1* expression (Supplementary Fig. S4). These data reveal that *SOAR1* is a salt- and cold-induced gene.

**Fig. 7:**
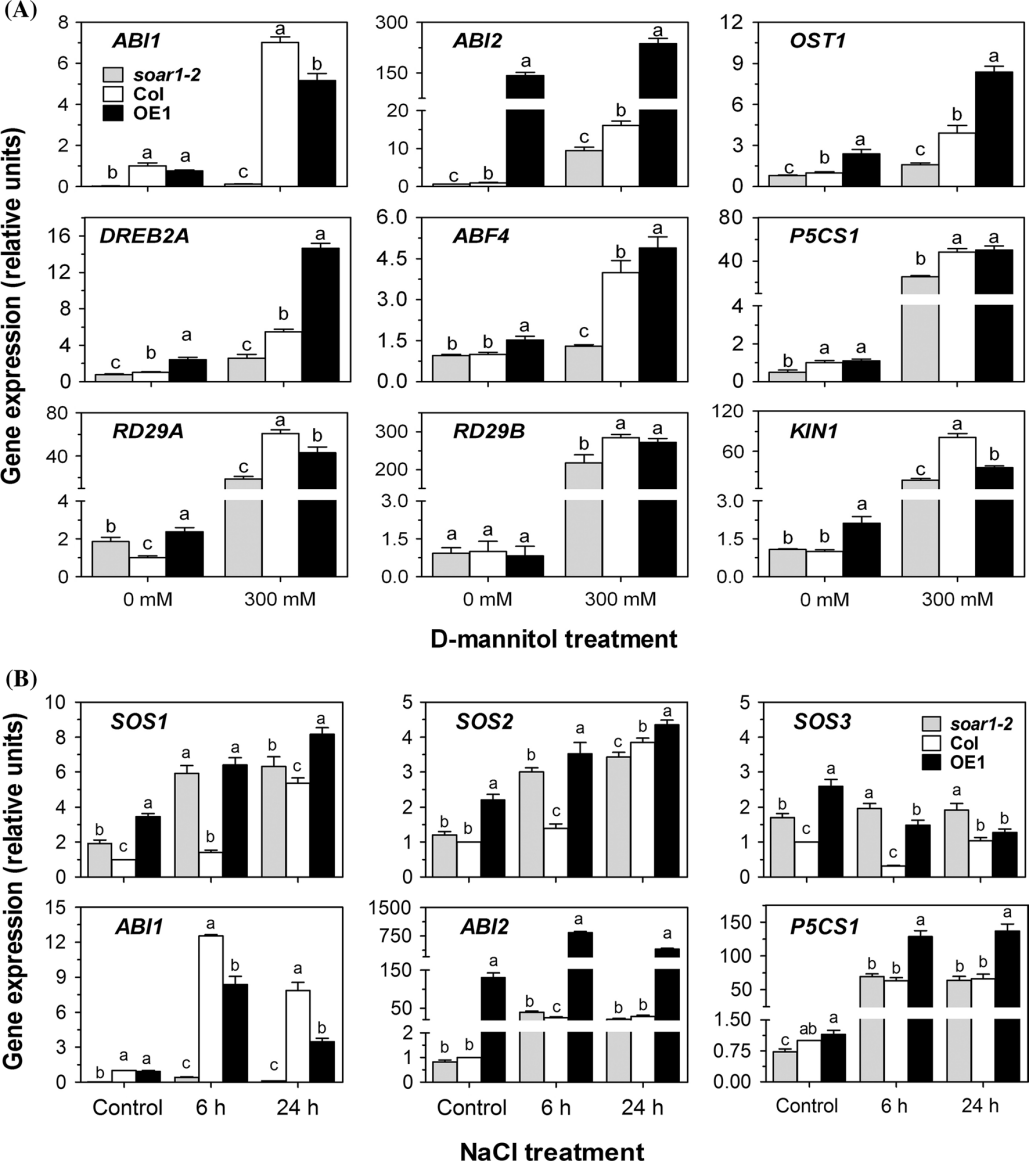
Changes in *SOAR1* expression alter expression of a subset of genes involved in osmotic and salt stress responses. The mRNA levels in the seedlings of wild-type Col, *soar1*-*2* mutant and the *SOAR1*-overexpression line OE1 were determined by real time RT-PCR. Two-week-old seedlings grown at 20 °C were treated with mannitol-free (0 mM) (as a control) or mannitol-containing (300 mM) solution for 24 h (**a**), or with the NaCl-free (0 mM) (as a control) or NaCl-containing (200 mM) solution for 6 or 24 h (**b**). The expression of the osmotic and salt stress responsive genes (as indicated in the figures) was analyzed. The gene expression levels were relative units normalized relative to the value from the sample of the wild-type Col plants (as 1). Each value is the mean ± SE of three independent determinations and different letters indicate significant differences at *P* < 0.05 (Duncan’s multiple range test) within the same treatment or at the same time point

The expression of *ABI2* was strongly induced by *SOAR1* overexpression (Fig. 7a, b), which confirms our previous observation (Mei et al. 2014), and the *ABI2* expression levels were even much higher in the *SOAR1* overexpression line than in the wild-type plants with salt and mannitol treatments (Fig. 7a, b). Both the salt and mannitol treatments promoted *ABI1* expression while its expression levels of both *soar1*-*2* and OE1 remained lower than those of wild type, noting scarcely detectable levels of the *ABI1* gene in the *soar1*-*2* mutant (Fig. 7a, b). The expression levels of the ABA-responsive gene *OST1* (Mustilli et al. 2002) and abiotic stress/ABA-responsive gene *DREB2A* (Liu et al. 1998), were repressed in the *soar1*-*2* mutant, while these two genes and the ABA-responsive genes *ABF4* (Choi et al. 2000), *RD29A* (Yamaguchi-Shinozaki and Shinozaki 1994) and *KIN1* (Gilmour et al. 1998) were up-regulated in the OE1 line (Fig. 7a). In response to the mannitol-induced osmotic stress, the expression of *OST1*, *DREB2A*, *ABF4*, *P5CS1*, *RD29A*, *RD29B* (Yamaguchi-Shinozaki and Shinozaki 1994), and *KIN1* was repressed in the *soar1*-*2* mutant, while that of *OST1*, *DREB2A* and *ABF4* was significantly up-regulated in the OE1 line (Fig. 7a). All these genes encodes positive ABA/stress-signaling regulators except for *ABI1* and *ABI2*, so these gene expression data, partly explaining the earlier-mentioned stress-response observations of the different genotypes, suggest a highly complex mechanism of osmotic/salt stress response by which SOAR1 functions.

Down- or up-regulation of *SOAR1* expression in the *soar1*-*2* mutant and the *SOAR1*-overexpression OE1 line, respectively, both enhanced the expression levels of *SOS1*, *SOS2* (Liu et al. 2000; Qiu et al. 2002; Shi et al. 2003) and *SOS3* (Liu and Zhu 1998) only with no statistically difference between the wild-type and *soar1*-*2* plants for *SOS3* with 24-h salt treatment (Fig. 7b), and the salt treatment promoted the expression levels of *SOS1* and *SOS2* in all the genotypes, noting a significant highest level of the two gene expression in the OE1 line with salt treatment of a 24-h duration (Fig. 7b). However, the salt treatment decreased the expression levels of *SOS3* in OE1 line with a transient decrease in wild-type and a slight stimulation in *soar1*-*2* (Fig. 7b). These data suggest that the positive regulatory roles of SOAR1 in response to salt stress is likely difficult to be fully explained by the SOS-mediated mechanism.

*P5CS1* is a gene involved in proline biosynthesis (Verbruggen et al. 1993; Kavi Kishor et al. 1995; Hare and Cress 1997), of which the expression levels increased with the mannitol and salt treatment, and the highest levels were observed in the *SOAR1* overexpression line with salt treatment (Fig. 7a, b). These findings are consistent with tolerance to salt stress of the *SOAR1* overexpression lines.

**Fig. 8:**
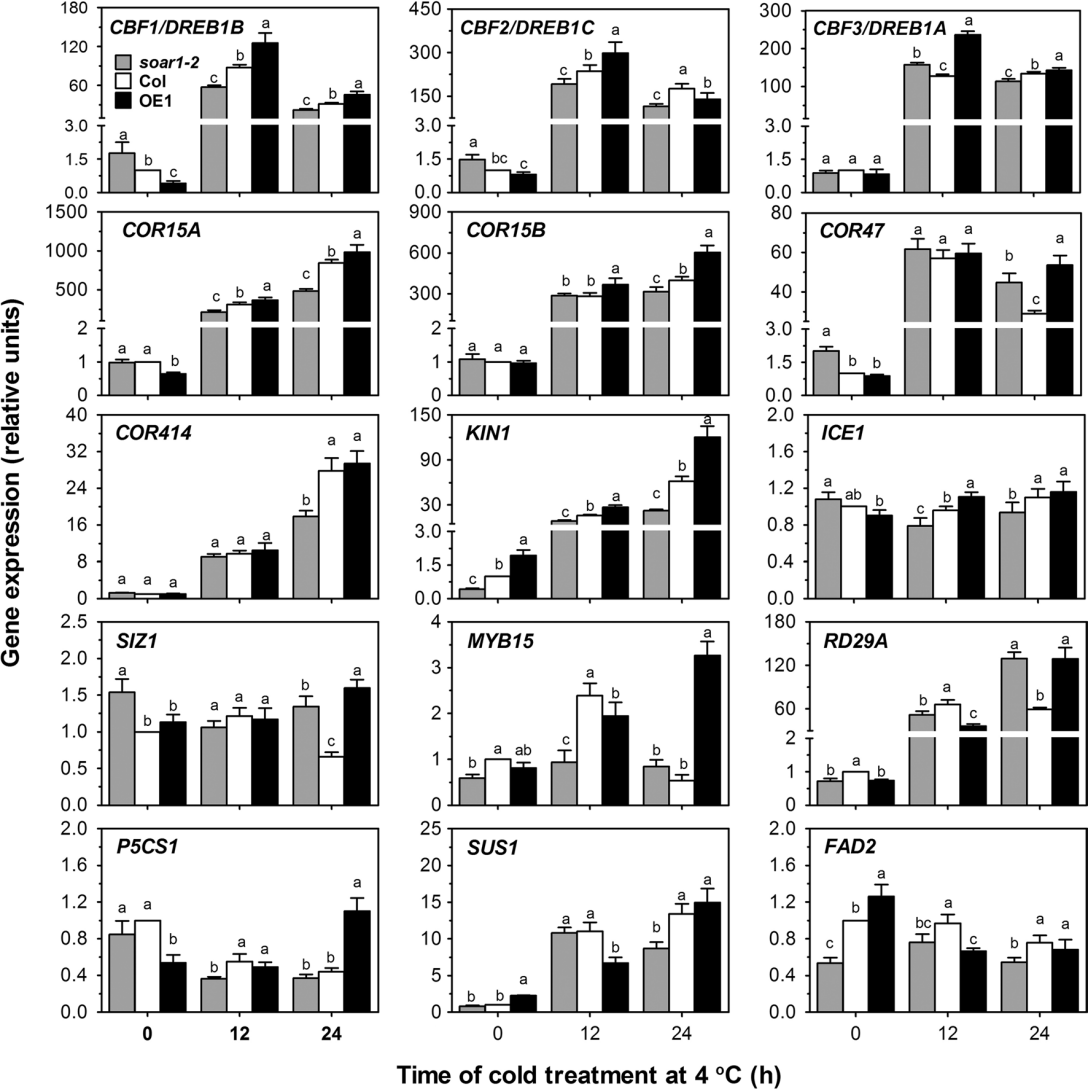
Changes in *SOAR1* expression alter expression of a subset of genes involved in cold stress response. The mRNA levels in the seedlings of wild-type Col, *soar1*-*2* mutant and the *SOAR1*-overexpression line OE1 were determined by real time RT-PCR. Seedlings were grown at 20 °C for 2 weeks, and transferred into a chamber at 4 °C for the indicated time. Expression of *CBF1/DREB1B*, *CBF2/DREB1C*, *CBF3/DREB1A*, *COR15A*, *COR15B*, *COR47*, *COR414*, *KIN1*, *ICE1*, *SIZ1*, *MYB15*, *RD29A*, *P5SC1*, *SUS*, and *FAD2* were analyzed. The gene expression levels were relative units normalized relative to the value from the sample of the wild-type Col plants (as 1). Each value is the mean ± SE of three independent determinations, and different letters indicate significant differences at *P* < 0.05 (Duncan’s multiple range test) within the same time point

The cold-responsive genes involved in the C-repeat binding factor/DRE-binding factor (CBF/DREB) transcriptional regulatory cascade, the best characterized cold-signalling pathway, were assayed after 4 °C treatment, which include *CBF1/DREB1B*, *CBF2/DREB1C*, *CBF3/DREB1A* (Yamaguchi-Shinozaki and Shinozaki 1994; Liu et al. 1998; Thomashow 1999), and CBF regulon genes *COR15A*, *COR15B*, *COR47*, *COR414*, *KIN1* (Gilmour et al. 1998), and *RD29A* (Yamaguchi-Shinozaki and Shinozaki 1994), as well as CBF upstream regulator-encoding genes *MYB15* (Agarwal et al. 2006)*, ICE1* (Chinnusamy et al. 2003) and *SIZ1* (Miura et al. 2007). Cold treatment increased the expression levels of *CBF1*, *CBF2* and *CBF3* with an attenuation transition from 12 h- to 24 h-treatment, noting higher levels in the OE1 and lower levels in the *soar1*-*2* mutant in most cases in comparison with wild type (Fig. 8, Supplementary Fig. S5). The expression profiles of the CBF downstream regulons genes *COR15A*, *COR15B*, *COR414* and *KIN1* were similar to those of the *CBF*s with a progressive increase instead of the attenuation transition from12 h- to 24 h-treatment for *CBF*s (Fig. 8, Supplementary Fig. S5). The data from these genes are globally consistent with the observation that SOAR1 positively regulates plant response to cold stress. The other two CBF downstream regulons genes *COR47* and *RD29A* were also induced by the cold treatment but with higher levels in both the *soar1*-*2* mutant and OE1 line by the 24 h-cold treatment. The cold-induced expression of the gene encoding an upstream, positive regulator of *CBF*s, ICE1, following an essentially similar profile to that of *CBF*s, could partly explain the expression profile of *CBF*s, but the expression of other two genes encoding the *CBF*-upstream, positive regulator SIZ1 and negative regulator MYB15, could not explain the expression profile of *CBF*s under the cold condition (Fig. 8).

Additionally, we tested three genes involved in metabolisms potentially to help cells to cope with cold stress. Globally, the *P5CS1* gene expression were not significantly induced by cold treatment in most cases, but showed a significantly high level in the *SOAR1*-overexpression OE1 line following a 24-h cold-treatment, which is consistent with the cold tolerance of the OE1. The expression of the *SUS1* gene, involved in solute production (Déjardin et al. 1999; Gilmour et al. 2000) like *P5CS1*, was strongly induced by the cold treatment; and the *FAD2* gene, positively involved in membrane stability (Miquel et al. 1993), was repressed by the cold treatment (Fig. 8). However, the levels of the two genes in the different genotypes (Fig. 8) could not explain the data of plant tolerance to cold stress (Fig. 6).

Previously, we observed that ABA concentration was not affected by down- or up-regulation of *SOAR1* expression (Mei et al. 2014). We tested further, in the present experiment, the expression of genes involved in ABA biosynthesis and catabolism. The tested ABA biosynthetic enzyme-encoding genes include *AAO1*, *AAO3*, *AAO4* (Sekimoto et al. 1998; Seo et al. 2000), *ABA1* (Duckham et al. 1991; Xiong et al. 2002), *ABA2* (Cheng et al. 2002; González-Guzmán et al. 2002), *ABA3* (Bittner et al. 2001; Xiong et al. 2001), *ABA4* (North et al. 2007), and *NCED3* (Iuchi et al. 2001). The tested ABA catabolic enzyme-encoding genes include *AtCYP707A1*, *AtCYP707A2*, *AtCYP707A3* (Kushiro et al. 2004; Saito et al. 2004), and *UGT71B6* (Lim et al. 2005; Priest et al. 2006). We observed that, in the germinating seeds, the expression of most ABA biosynthetic and catabolic genes was not significantly changed in the OE1 line, while in the *soar1*-*2* mutant, the expression of most of both the ABA biosynthetic and catabolic enzyme-encoding genes was up-regulated as compared with the wild-type plants (Supplementary Fig. S6), suggesting that, in the *soar1*-*2* mutant, both ABA biosynthetic and catabolic processes are likely to be promoted. With exogenous ABA treatment, differences in the expression levels of these genes were attenuated between the *soar1*-*2* mutant and the wild-type plants, while in OE1, ABA-induced effects of these genes observed in wild-type plants decreased (Supplementary Fig. S6). Overall, these data suggest that changes in SOAR1 expression may trigger a mechanism to balance ABA biosynthetic and catabolic processes, globally consistent with the endogenous ABA concentrations, which do not significantly differ among different genotypes as described previously (Mei et al. 2014).

## Discussion

### SOAR1 is a crucial, positive regulator of plant response to drought, salt and cold stresses, and likely to be useful in crop improvement

We provide genetic evidence that downregulation of *SOAR1* expression reduces, but upregulation of *SOAR1* expression enhances, ABA sensitivity in ABA-induced promotion of stomatal closure and inhibition of stomatal opening, and plant tolerance to multiple, major abiotic stresses including drought, high salinity and low temperature (Figs. 1, 2, 3, 4, 5, 6, Supplementary Figs. 1 and 2), demonstrating that SOAR1 is a crucial, positive regulator of plant response to abiotic stresses. Whereas several mitochondrial/chloroplast PPR proteins have been reported to regulate plant response to abiotic stresses (Zsigmond et al. 2008; Liu et al. 2010; Laluk et al. 2011; Murayama et al. 2012; Yuan and Liu 2012; Zhu et al. 2014), SOAR1 is the first cytosol-nucleus dual-localized protein, to our knowledge, to be identified as a crucial player in plant response to multiple, major abiotic stresses. The discovery of a cytosolic-nuclear PPR protein to regulate plant stress signaling suggest that PPR proteins may play roles in RNA processing not only in the organelles mitochondrion and chloroplast to regulate ROS homeostasis of cells, but also in the nucleus to modulate a wide range of cellular signaling processes in response to environmental cues of stresses.

Interestingly and importantly, the *SOAR1*-overexpression lines display strong abilities to tolerate drought, salt and cold stresses, with surprisingly high resistance to salt stress in germination and postgermination growth of seeds that are able to potentially germinate in seawater (Figs. 3, 4, Supplementary Figs. 1 and 2), while no negative impact of *SOAR1*-overexpression on plant growth and development was observed (Figs. 1, 3, 5). SOAR1 is highly conserved and has homologues in different plant species such as in *Vitis vinifera*, *Ricinus communis, Populus trichocarpa*, *Sorghum bicolor*, and *Oryza sativa* (Supplementary Fig. S7). Therefore, the *SOAR1* gene may likely be useful for improvement of crops by transgenic manipulation to enhance crop productivity in stressful conditions.

### How does SOAR1 function in response to abiotic stresses?

ABA is a central stress signal that has been believed to mainly modulates both water balance and osmotic stress/cellular dehydration tolerance to regulate plant adaptation to water deficit and salt stress, where the water balance is mainly controlled through guard cell regulation, the dehydration tolerance is dependent of osmosis-regulation proteins in all cells (Shinozaki et al. 2003; Zhu 2002, 2003). We previously showed that SOAR1 is a negative regulator of ABA signaling in seed germination and postgermination growth (Mei et al. 2014; Jiang et al. 2014). Unexpectedly, in the present experiment, we showed that SOAR1 is positively, but not negatively, involved in ABA-induced promotion of stomatal closure and inhibition of stomatal opening (Fig. 1), which explain in part enhanced tolerance of the *SOAR1*-overexpression plants to drought stress (Fig. 1). Additionally, the ability to significantly tolerate drought of these *SOAR1*-overexpression plants may also be linked to their tolerance to osmotic stress.

It is well known that ABA accumulates in salt stress as in other abiotic stresses, and increased levels of ABA result in inhibition of seed germination and is required for tolerance of seedling growth to salt (Zhu 2002, 2003; Shinozaki et al. 2003). We previously showed that down-expression of *SOAR1* increases, but up-expression of *SOAR1* dramatically decreases ABA sensitivity in ABA-induced seed-germination inhibition and postgermination-growth arrest (Mei et al. 2014; Jiang et al. 2014), and the *SOAR1*-overexpressiing seeds germinate even in 500 μM-(±)ABA-containing medium (Jiang et al. 2014). Therefore, the hypersensitivity resulting from *SOAR1* down-expression and resistance against high salinity from *SOAR1* up-expression in seed germination and postgermination growth (Figs. 2, 3, 4, Supplementary Figs. 1 and 2) should be attributed to ABA hypersensitivity of the *SOAR1* down-expressing mutants and strong ABA insensitivity of the *SOAR1* over-expressing lines (see Mei et al. 2014; Jiang et al. 2014) in the situation of the salt-induced high levels of ABA. This point of view may be supported by the observations that the *ABI2*-overexpressing line, highly insensitive to ABA (Sun et al. 2011; Mei et al. 2014), tolerates, but *abi1 abi2* double knockout mutant, overly sensitive to ABA, is hypersensitive, to salt stress in seed germination and postgermination growth, as observed in this experiment (Figs. 2, 3, 4, Supplementary Figs. 1 and 2). The same phenomenon was also observed previously (Achard et al. 2006). The mannitol-induced osmosis-related phenotypes of the *SOAR1*-down- or—over-expressing plants in seed germination and postgermination growth may be also explained by changes in ABA sensitivity of these genotypes (Fig. 2). Interestingly and importantly, however, the mature plants of the *SOAR1*-over-expressing lines, similar to those of the *abi1 abi2* double knockout mutant, tolerate, but the mature plants of the *SOAR*-down-expressing mutants, similar to those of the *ABI2*-overexpressing line, are overly sensitive to, salt stress (Fig. 5), which reveals that SOAR1 positively, but ABI2 negatively, regulates plant response to salt stress in seed germination and seedling growth, whereas both are negative regulators of ABA signaling in these two developmental processes. Therefore, unlike the *ABI2*-overexpression line, the tolerance to salt stress of the *SOAR1*-overexpression lines in postgermination growth especially during a prolonged period (18 days after stratification, Fig. 3), observed when their seeds were directly sown in ABA-containing medium, should be ascribed not only to its negative role of SOAR1 in ABA signaling in seed germination (resulting in strong ABA insensitivity of the these lines) but also to its positive role in response to salt stress.

Plant response to cold stress, like that to drought and salt stresses, requires both ABA-dependent and -independent signaling pathways (Shinozaki et al. 2003; Zhu 2002, 2003; Qin et al. 2011; Ma and Qi 2014; Shi and Yang 2014). The CBF-mediated signaling pathway has been believed to be an ABA-independent signaling pathway (Yamaguchi-Shinozaki and Shinozaki 1994; Liu et al. 1998; Thomashow 1999; Gilmour et al. 1998; Chinnusamy et al. 2003, 2007; Agarwal et al. 2006; Miura et al. 2007; Lata and Prasad 2011; Shi and Yang 2014). Like its positive role in plant response to drought and salt stresses as mentioned above, SOAR1 positively regulates plant response to cold stress (Fig. 6), functioning at least partly through the CBF signaling pathway, as evidenced by the analysis of gene expression in the *SOAR1*-down- and over-expressing plants (Fig. 8).

Taken together, all these findings reveal that SOAR1, a cytosolic-nuclear PPR protein, plays crucial roles in plant response to multiple, major abiotic stresses, likely integrating ABA-dependent and independent pathways. The SOAR1-mediated ABA signaling, functioning positively in ABA-induced inhibition of seed germination and postgermination growth arrest but negatively in ABA-induced promotion of stomatal closure and inhibition of stomatal opening, is likely different from the ABI2/ABI1 PP2C-mediated signaling downstream of PYR/PYL-RCAR receptors for ABA (Mustilli et al. 2002; Yoshida et al. 2006; Fujii et al. 2009; Ma et al. 2009; Park et al. 2009; Santiago et al. 2009; Umezawa et al. 2009; Vlad et al. 2009, 2010; Nishimura et al. 2010; Cutler et al. 2010). SOAR1 has been identified as a downstream player of CHLH/ABAR, a candidate ABA receptor that positively regulates ABA signaling (Shen et al. 2006; Legnaioli et al. 2009; Wu et al. 2009; Shang et al. 2010; Jia et al. 2011; Jiang et al. 2011; Tsuzuki et al. 2011, 2013; Du et al. 2012; Liu et al. 2012; Xu et al. 2012; Liu et al. 2013; Yan et al. 2013; Mei et al. 2014; Jiang et al. 2014; Zhang et al. 2013, 2014; Wang and Zhang, 2014). Elucidation of the mechanism by which SOAR1 functions, especially in the nuclear events, will be of great importance to help to understand highly complicated ABA and stress signalling network.

## Electronic supplementary material

Supplementary material 1 (PDF 2140 kb)
