# Supplementary material for: Crucial roles of the pentatricopeptide repeat protein SOAR1 in Arabidopsis response to drought, salt and cold stresses
Source: Plant Mol Biol. 2015 Jun 21;88(4-5):369–85. doi: 10.1007/s11103-015-0327-9 (PMC4486114; doi:10.1007/s11103-015-0327-9)
Supplement: Supplementary file 1 — Supplementary material 1 (PDF 2140 kb) [file 11103_2015_327_MOESM1_ESM.pdf]

Supplementary Data

Jiang *et al*., Crucial roles of the pentatricopeptide repeat protein SOAR1 in *Arabidopsis* response to drought, salt and cold stresses

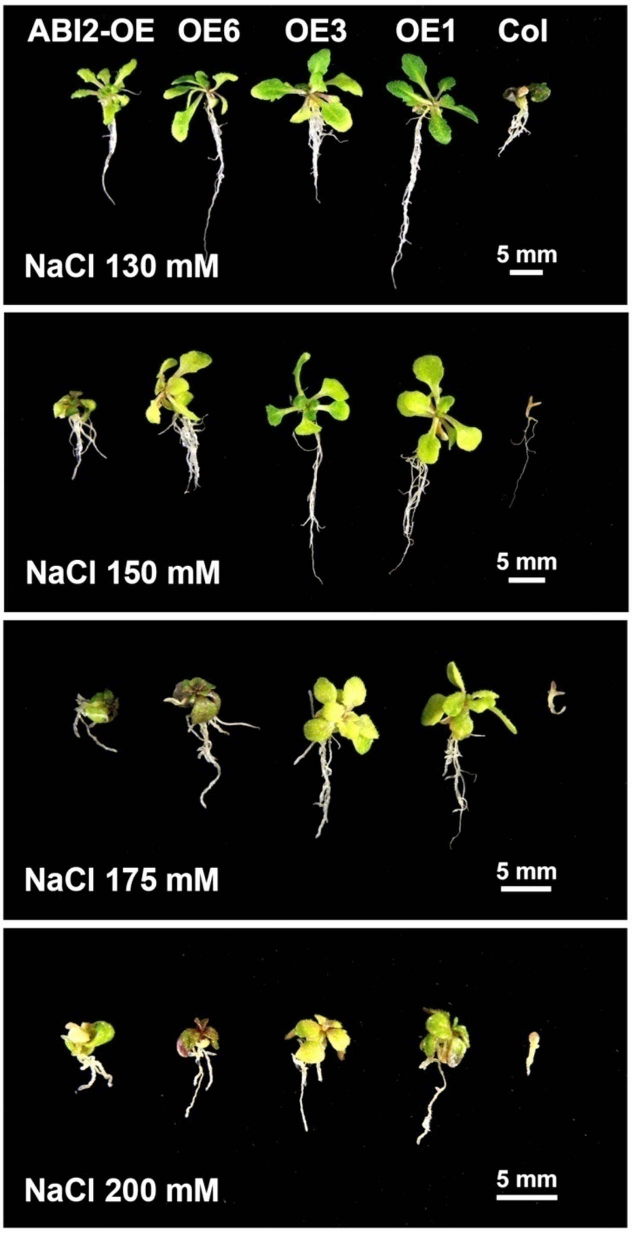

Fig. S1. Postgermination growth of the *SOAR1*-overexpression lines under salt stress. Postgermination growth of the wild-type Col, three *SOAR1*-overexpression lines (OE1, OE3 and OE6) and an *ABI2*-overexpression line (ABI2-OE) in the MS medium containing, 130, 150, 175, and 200 mM NaCl, was recorded 18 d after stratification. The entire experiment was replicated three times with similar results. Bars indicate 5 mm.

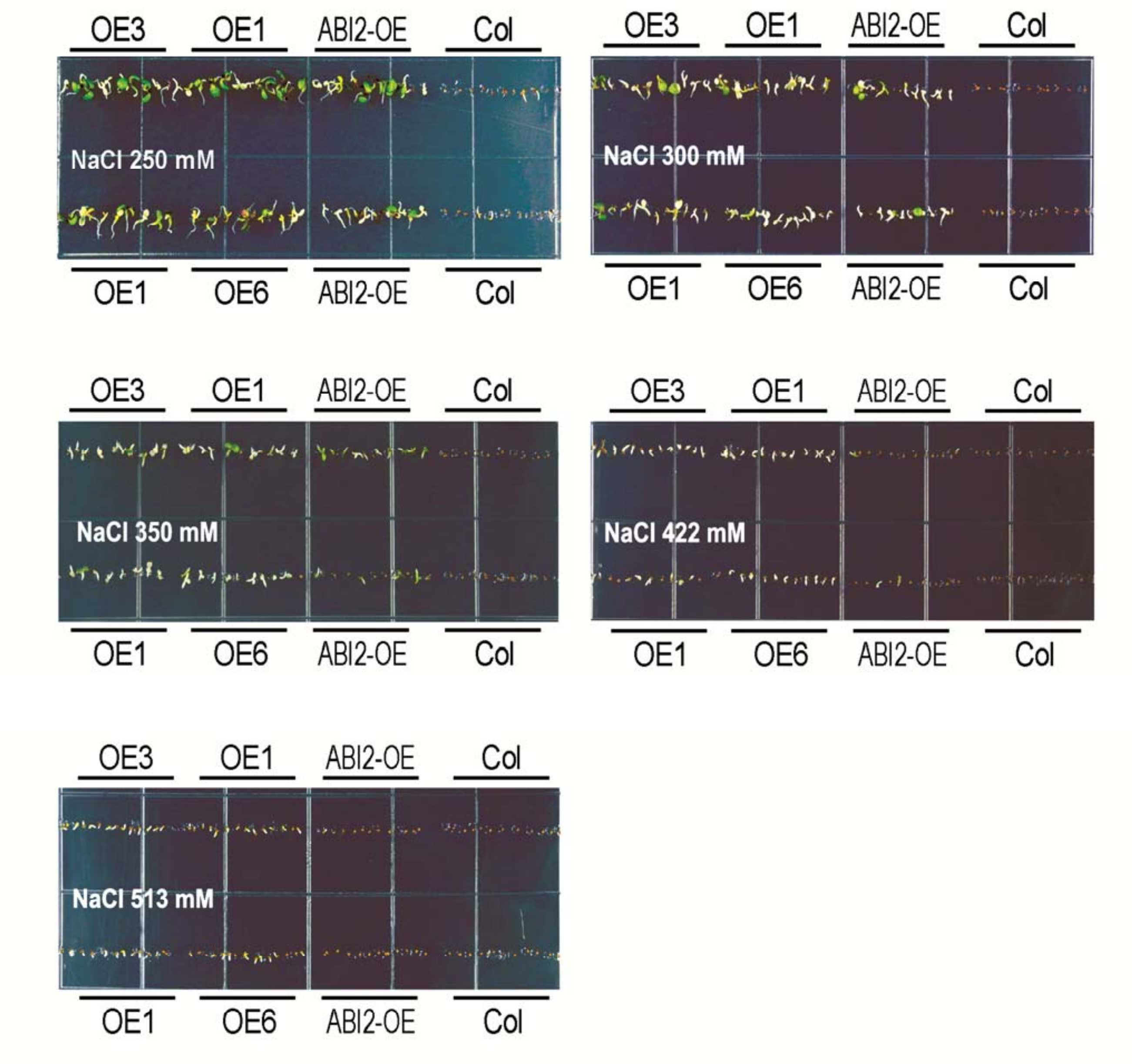

Fig. S2. Test of the extremity of the NaCl concentrations under which seeds of the *SOAR1*-overexpression lines germinate and continue to grow (pictures in plates). Germination and postgermination growth of the wild-type Col, an *ABI2*-overexpression line (ABI2-OE) and three *SOAR1*-overexpression lines OE1, OE3 and OE6 in the MS medium containing 250, 300, 350, 422, and 513 mM NaCl, were investigated two weeks after stratification. The entire experiment was replicated three times with similar results.

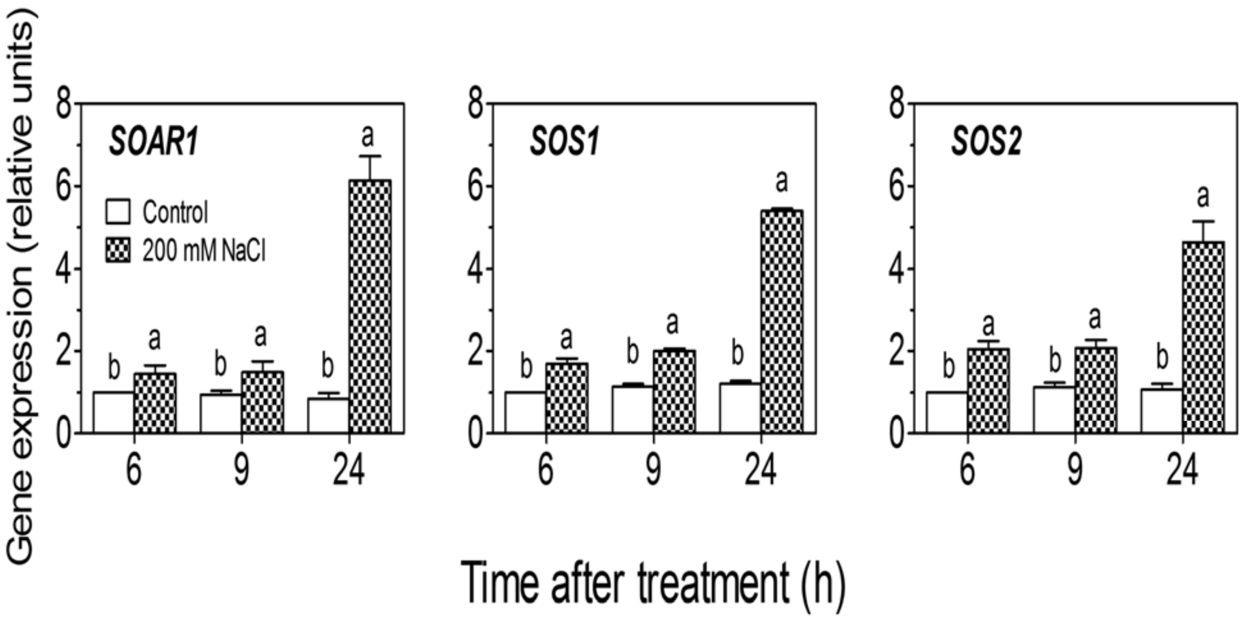

Fig. S3. Expression of *SOAR1* under salt stresses. RNA samples were prepared from the 2-week-old wild-type Col seedlings treated with the NaCl-free (control) or NaCl-containing (200 mM) medium at the indicated time after the treatments. Expression levels of *SOAR1* were analyzed by quantitative real-time PCR. The gene expression levels are presented as relative units normalized relative to the value from the sample of the wild-type Col plants treated with the control solution after the treatments. Each value is the mean ±SE of three independent determinations, and different letters indicate significant differences at $P<0.05$ (Duncan's multiple range test) within the same time point.

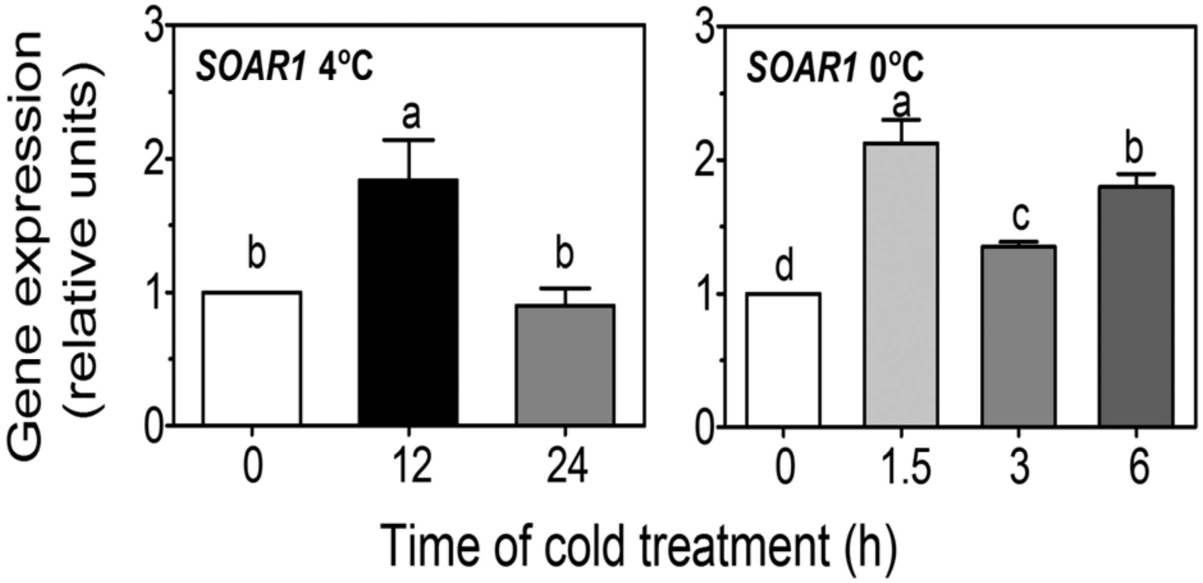

Fig. S4. Expression of *SOAR1* under cold stress. RNA samples were prepared from the 2-week-old wild-type Col seedlings grown under cold conditions at 4 °C or 0 °C at the indicated time point. Gene expression was analyzed by quantitative real-time PCR, of which the levels were relative units normalized relative to the value from the sample of the wild-type Col plants at the <0> time point (as 1). Each value is the mean ±SE of three independent determinations, and different letters indicate significant differences at *P*<0.05 (Duncan's multiple range test).

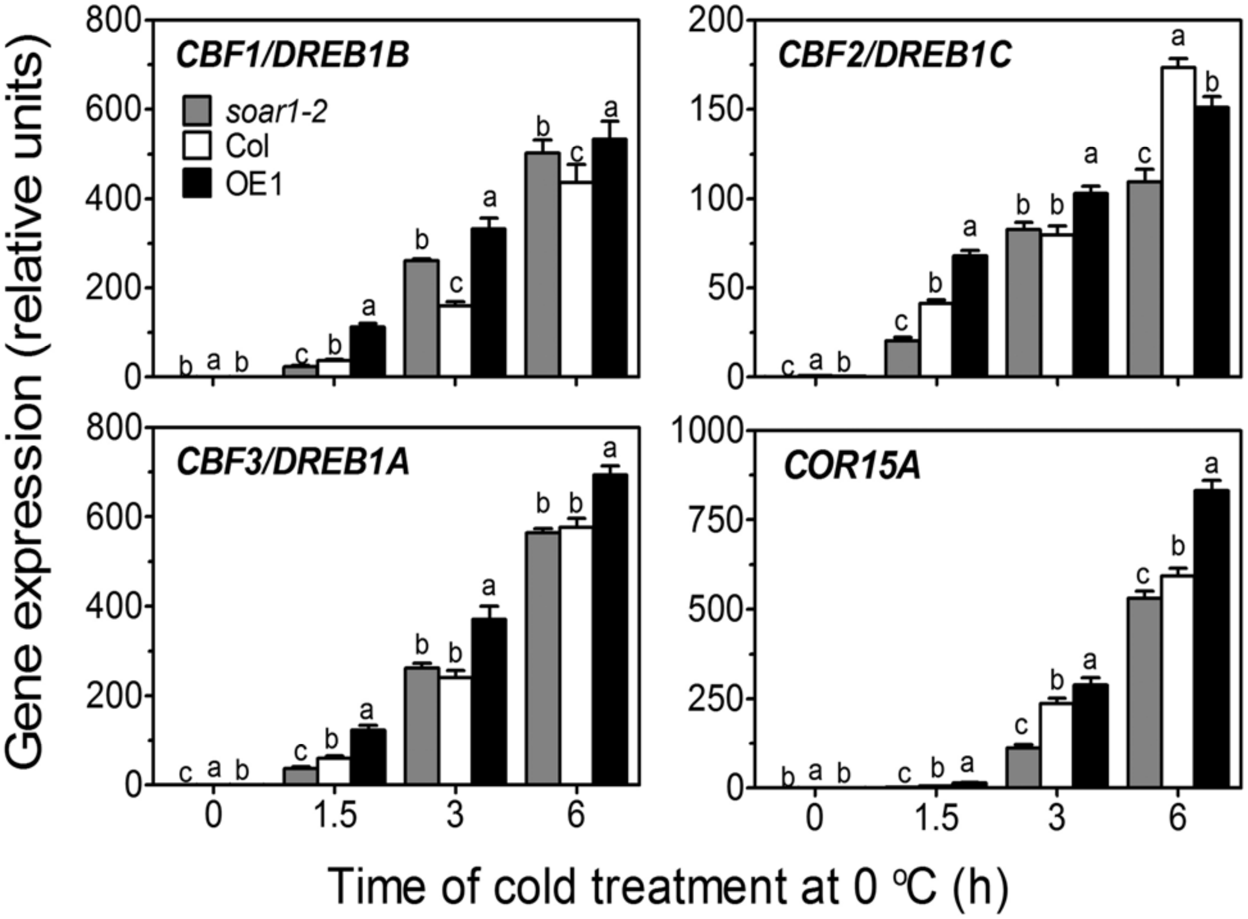

Fig. S5. Changes in *SOAR1* expression alter expression of *CBFs* and *COR15A* under cold condition. The 2-week-old seedlings of wild-type Col, *soar1-2* mutant and a *SOAR1*-overexpression line OE1 were subjected to cold treatment at 0 °C, and sampled for quantitative real-time PCR analysis at the indicated time points after the treatment. The gene expression levels were relative units normalized relative to the value from the sample of the wild-type Col plants (as 1). Each value is the mean ±SE of three biological determinations, and different letters indicate significant differences at *P*<0.05 (Duncan's multiple range test) within the same time point.

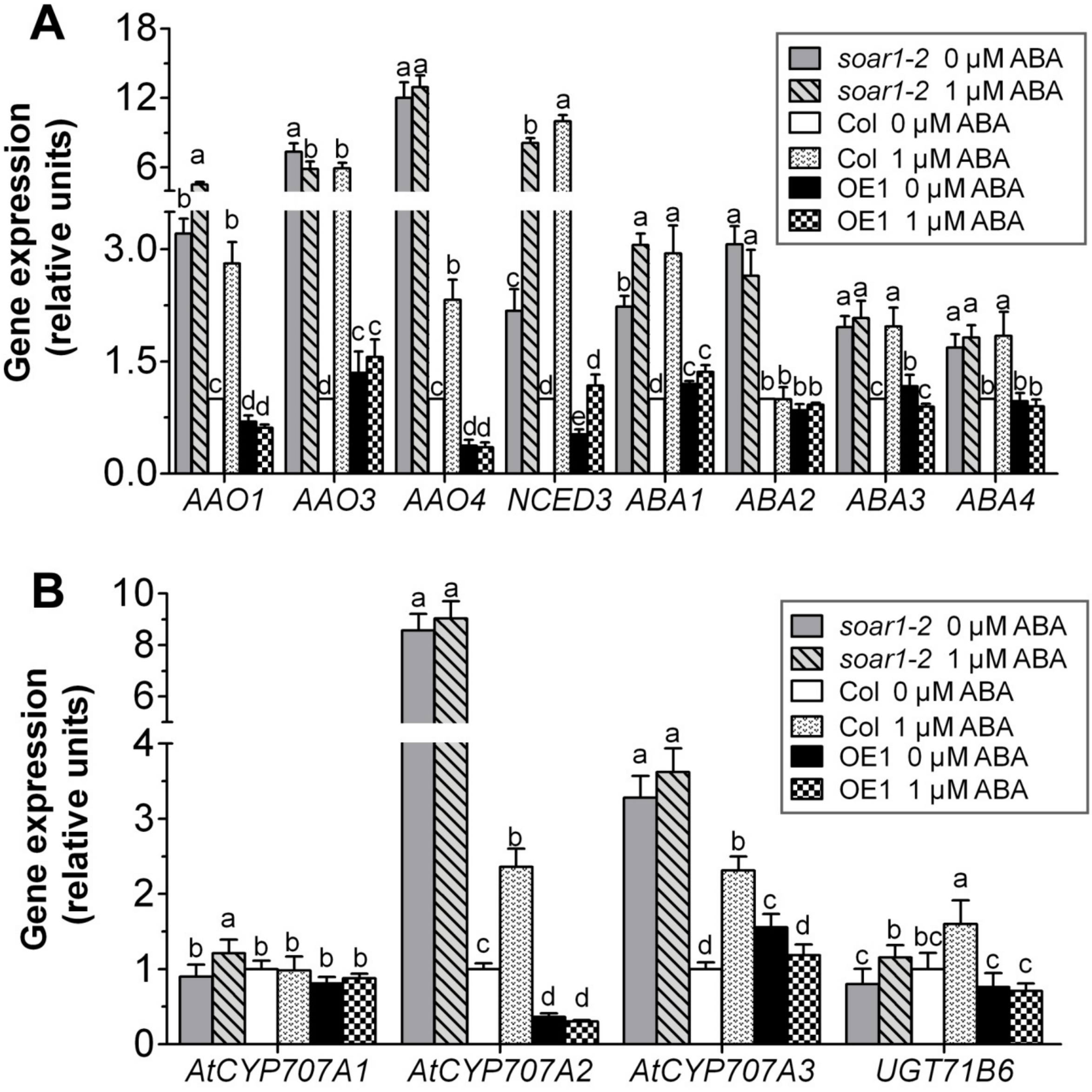

Fig. S6. Changes in *SOAR1* expression alter expression of a subset of ABA biosynthesis- and catabolism-related genes. RNA sample was extracted from the germinating seeds of different genotypes treated with the ABA-free (0 μM) or 1 μM-ABA-containing solution 24 h after three-day stratification, and the gene expression levels of ABA biosynthetic enzyme-encoding genes *AAO1*, *AAO3*, *AAO4*, *ABA1-4* and *NCED3*, as well as ABA catabolic enzyme-encoding genes *AtCYP707A1*, *AtCYP707A2*, *AtCYP707A3* and *UGT71B6* were analyzed by quantitative real-time PCR. The gene expression levels were relative units normalized relative to the value from the sample of the wild-type Col plants (as 1). Each value is the mean ±SE of three biological determinations, and different letters indicate significant differences at $P<0.05$ (Duncan's multiple range test) among the treatments within the same gene.

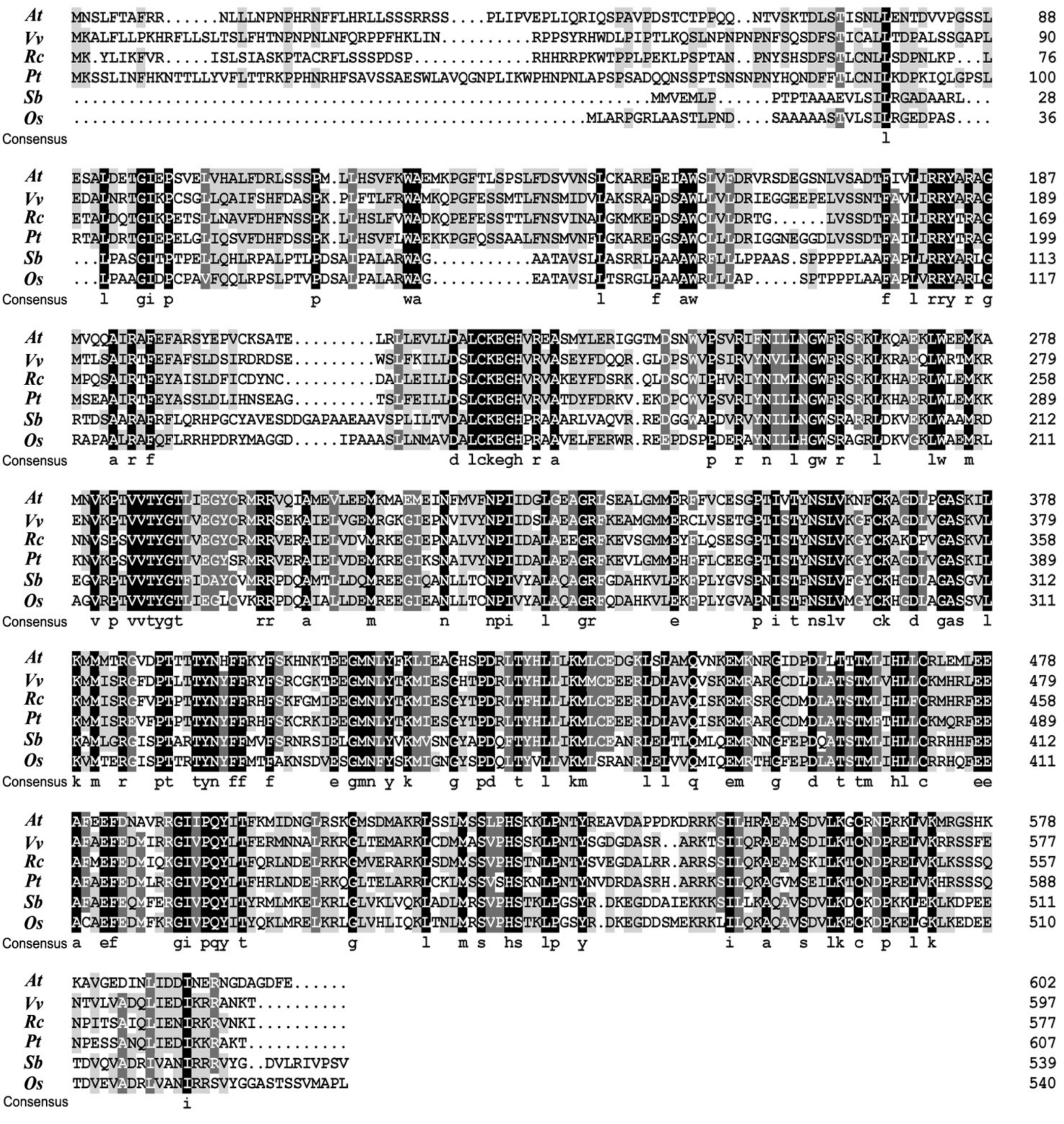

Fig. S7. Alignment of amino acid sequence of SOAR1 in different plant species. The homologous sequence was aligned by ClustalX2 (http://www.clustal.org/). The abbreviation of plant species: *At*, *Arabidopsis thaliana; Vv*, *Vitis vinifera*; *Rc*, *Ricinus communis*; *Pt*, *Populus trichocarpa*; *Sb*, *Sorghum bicolor*; *Os*, *Oryza sativa*. The black background represents the identical amino acid residues, and the dark grey background represents the similarity more than 75%, and the light grey background represents the similarity more than 50%. The numbers in the left of the sequence indicate the number of predicted amino acid residues. The dot represents the gap in order to maximize alignment.

Table S1. Primers for quantitative real-time PCR used in this study.

Abbreviations: for, forward primer; rev, reverse primer

Gene	Primer sequence
***Actin2/8* for**	5’-GGTAACATTGTGCTCAGTGGTGG-3’
***Actin2/8* rev**	5’-AACGACCTTAATCTTCATGCTGC-3’
***SOAR1* for**	5’-TACGGAACTTAGGTTGCTTGAG-3’
***SOAR1* rev**	5’-AACAACAGTCGGCTTCACATTC-3’
***ABI1* for**	5’-AGAGTGTGCCTTTGTATGGTTTTA-3’
***ABI1* rev**	5’-CATCCTCTCTCTACAATAGTTCGCT-3’
***ABI2* for**	5’-GATGGAAGATTCTGTCTCAACGATT-3’
***ABI2* rev**	5’-GTTTCTCCTTCACTATCTCCTCCG-3’
***RD29A* for**	5’-ATCACTTGGCTCCACTGTTGTTC-3’
***RD29A* rev**	5’-ACAAAACACACATAAACATCCAAAGT-3’
***RD29B* for**	5’-CTTGGCACCACCGTTGGGACTA-3’
***RD29B* rev**	5’-TCAGTTCCCA GAATCTTGAACT-3’
***P5CS1* for**	5’-CGACATGGGAGTGCACACACAGAC-3’
***P5CS1* rev**	5’-CCTCTCATTATCCATCTCGTTG-3’
***SOS1* for**	5’-GGCAGCATGGTTAATGTGTAC-3’
***SOS1* rev**	5’-TTCATCTTCTTCATCGCTTG-3’
***SOS2* for**	5’-ATTTGACAGGCGACAGGATTTTG-3’
***SOS2* rev**	5’-GCCACCTCGTAAATCTCTATC-3’
***SOS3* for**	5’-TCATCGAGCGAGAAGAATTG-3’
***SOS3* rev**	5’-CGTCCTATTTATGTCCTTTAG-3’
***OST1* for**	5’-GATCCCGAGGAACCAAAGAA-3’
***OST1* rev**	5’-ATCCTCTTTGCAGGGTCAGC-3’
***ABF4* for**	5’-AACAACTTAGGAGGTGGTGGTC-3’
***ABF4* rev**	5’-CTTCAGGAGTTCATCCATGTTC-3’
***DREB2A* for**	5’-AAGGTAAAGGAGGACCAGAG-3’
***DREB2A* rev**	5’-ACACAACCAGGAGTCTCAAC-3’
***CBF1/DREB1B* for**	5’-GCATGTCTCAACTTCGCTGA-3’
***CBF1/DREB1B* rev**	5’-ATCGTCTCCTCCATGTCCAG-3’
***CBF2/DREB1C* for**	5’-TGACGTGTCCTTATGGAGCTA-3’
***CBF2/DREB1C* rev**	5’-CTGCACTCAAAAACATTTGCA-3’
***CBF3/DREB1A* for**	5’-GATGACGACGTATCGTTATGGA-3’
***CBF3/DREB1A* rev**	5’-TACACTCGTTTCTCAGTTTTACAAAC-3’
***COR15A* for**	5’-GCTTCAGATTTCGTGACGGATAAAAC-3’
***COR15A* rev**	5’-GCAAAACATTAAAGAATGTGACGGTG-3’
***COR15B* for**	5’-AAAGCAGAGTGGTGTTGGTACCGT-3’
***COR15B* rev**	5’-TCATCGAGGATGTTGCCGTCACTT-3’
***COR47* for**	5’-CAGTGTCGGAGAGTGTGGTG-3’
***COR47* rev**	5’-ACAGCTGGTGAATCCTCTGC-3’
***COR414* for**	5’-AGCATTCTTGGCGCTTATTGCTCG-3’
***COR414* rev**	5’-TCCCTGCTCGTGAGAAATGTTGGA-3’
***ICE1* for**	5’-TCCTAAAGGCCAGCAAGCTA-3’

Gene	Primer sequence
***ICE1* rev**	5’-CTCTTGTCCTTCTTGGCATTG-3’
***SIZ1* for**	5’-ATAGCGCCTCTGGGAATCAT-3’
***SIZ1* rev**	5’-GCCTTGTCTTGTCTACTGTCATTCATAC-3’
***MYB15* for**	5’-GGTGCGGATATCGATGAAAG-3’
***MYB15* rev**	5’-CATTATTAGCGGAGCCCAAG-3’
***FAD2* for**	5’-CCAAAGCAGAAATCAGCAATCA-3’
***FAD2* rev**	5’-GCAGCAGCGTAACGGTAAAGAC-3’
***KIN1* for**	5’-TGGAGCTGGAGCACAACA-3’
***KIN1* rev**	5’-GACCCGAATCGCTACTTGTTC-3’
***SUS1* for**	5’-TTCTGGAAGCATGTCTCGAA-3’
***SUS1* rev**	5’-GTGCAAGAGGAACAGCCTGA-3’
***ABA1* for**	5’-TCACTGACAAAGCCGATGACC-3’
***ABA1* rev**	5’-CGAAACGCAACAATCGTCG-3’
***ABA2* for**	5’-AAGCATGAAACATGCAGCTCG-3’
***ABA2* rev**	5’-AAGAATGTGGACCAACGCCTC-3’
***ABA3* for**	5’-TAGTGAAGAAATCCAATGAGCCCT-3’
***ABA3* rev**	5’-CTTCTTCCCCAACTCCAATCCAA-3’
***ABA4* for**	5’-GACTCTTGCTTCTGCTTGGATTC-3’
***ABA4* rev**	5’-AGAAGGCAAAGTGAAACCGAATG-3’
***NCED3* for**	5’-AGCCGCCATTATCGTCTTCTC-3’
***NCED3* rev**	5’-GGAGTGTGAAGCGCAGATGAA-3’
***AAO1* for**	5’-TGCTCTGTTTTTGTTTGTGGTTTG-3’
***AAO1* rev**	5’-CACAATCCATTGCCATCGACAT-3’
***AAO3* for**	5’-TCCATCATGGACTGCTCCTTC-3’
***AAO3* rev**	5’-CGAGACACTAGCGCCAAGAAA-3’
***AAO4* for**	5’-CAACAGTGGTCATCACAAGAACC-3’
***AAO4* rev**	5’-GGAACCGGTAACTCAAAACCAAG-3’
***AtCYP707A1* for**	5’-CAAAACCCAATACGTTCATGCCA-3’
***AtCYP707A1* rev**	5’-CTCGCTCCAACAATTGACCAAAC-3’
***AtCYP707A2* for**	5’-CAAGAAAAATGCCACTGACCACT-3’
***AtCYP707A2* rev**	5’-TCCAACCCTTTGGGATCAAGTAG-3’
***AtCYP707A3* for**	5’-TCTCAATACTCGGCAAAGACGAA-3’
***AtCYP707A3* rev**	5’-GAGCTTTCATGGCTTTGTGGAAT-3’
***UGT71B6* for**	5’-GGTTCAGTGAGGAACAAGTGAGA-3’
***UGT71B6* rev**	5’-TAACCTTTCCTCTGTTAGCCGTC-3’

Table S2. Duncan's multiple range tests for the data of the water loss rate presented in Fig. 1B. Different letters indicate significant differences at $P<0.05$.

**Time (h)**	**Genotype**	**Mean**	**Significance of difference 0.05**	**Time (h)**	**Genotype**	**Mean**	**Significance of difference 0.05**
**1**	Col	5.3	**c**	**2**	Col	8.9	**d**
	soar1-2	6.8	**c**		*soar1-2*	11.8	**c**
	soar1-3	10.2	**b**		*soar1-3*	16.9	**b**
	OE1	4.5	**cd**		OE1	7.7	**e**
	OE6	3.5	**d**		OE6	6.4	**e**
	ABI2-OE	16.4	**a**		ABI2-OE	28.7	**a**
**3**	Col	13.1	**d**	**4**	Col	16.4	**d**
	soar1-2	16.9	**c**		*soar1-2*	21.8	**c**
	soar1-3	25.8	**b**		*soar1-3*	32.9	**b**
	OE1	11.6	**e**		OE1	15.4	**de**
	OE6	9.8	**e**		OE6	12.4	**e**
	ABI2-OE	38.6	**a**		ABI2-OE	50.4	**a**
**5**	Col	19.3	**d**	**6**	Col	23.1	**d**
	soar1-2	26.1	**c**		*soar1-2*	30.1	**c**
	soar1-3	37.6	**b**		*soar1-3*	42.9	**b**
	OE1	17.9	**de**		OE1	20.8	**de**
	OE6	15.2	**e**		OE6	17.4	**e**
	ABI2-OE	56.8	**a**		ABI2-OE	62.9	**a**
**7**	Col	26.2	**d**	**8**	Col	29.1	**d**
	soar1-2	34.5	**c**		*soar1-2*	38.5	**c**
	soar1-3	48.4	**b**		*soar1-3*	52.4	**b**
	OE1	24.5	**de**		OE1	27.8	**de**
	OE6	20.4	**e**		OE6	22.9	**e**
	ABI2-OE	67.8	**a**		ABI2-OE	69.7	**a**

Table S3. Duncan's multiple range tests for the data of the seed germination rateof the controls presented in Fig. 2A and 2C. Different letters indicate significant differences at *P*<0.05.

**Time (h)**	**Genotype**	**Mean**	**Significance of difference 0.05**	**Time (h)**	**Genotype**	**Mean**	**Significance of difference 0.05**
	Col	0	**a**		Col	8.1	**d**
	soar1-2	0	**a**		*soar1-2*	0	**e**
	soar1-3	0	**a**		*soar1-3*	0	**e**
**0**	OE1	0	**a**	**24**	OE1	20.1	**b**
	OE3	0	**a**		OE3	15.8	**c**
	OE6	0	**a**		OE6	55.7	**a**
	ABI2-OE	0	**a**		ABI2-OE	7.8	**d**
	Col	81.6	**b**		Col	95	**a**
	soar1-2	48.8	**c**		*soar1-2*	91.4	**b**
	soar1-3	42.7	**d**		*soar1-3*	76.3	**c**
**36**	OE1	91.2	**a**	**48**	OE1	95	**a**
	OE3	80	**b**		OE3	95.8	**a**
	OE6	92.3	**a**		OE6	96.1	**a**
	ABI2-OE	83.4	**b**		ABI2-OE	93	**b**
	Col	97.9	**ab**		Col	98.9	**a**
	soar1-2	97.6	**ab**		*soar1-2*	98.4	**a**
	soar1-3	97.2	**ab**		*soar1-3*	99. 1	**a**
**60**	OE1	97	**ab**	**72**	OE1	98.3	**a**
	OE3	97.5	**ab**		OE3	98.3	**a**
	OE6	98.1	**a**		OE6	99.1	**a**
	ABI2-OE	96.5	**b**		ABI2-OE	98.2	**a**
	Col	100	**a**		Col	100	**a**
	soar1-2	100	**a**		*soar1-2*	100	**a**
	soar1-3	100	**a**		*soar1-3*	100	**a**
**96**	OE1	100	**a**	**120**	OE1	100	**a**
	OE3	100	**a**		OE3	100	**a**
	OE6	100	**a**		OE6	100	**a**
	ABI2-OE	100	**a**		ABI2-OE	100	**a**

Table S4. Duncan's multiple range tests for the data of the seed germination rates of the D-mannitol (300 mM) treatment presented in Fig. 2A. Different letters indicate significant differences at *P*<0.05.

Time (h)	Genotype	Mean	Significance of difference 0.05	Time (h)	Genotype	Mean	Significance of difference 0.05
	Col	0	**a**		Col	0	**a**
	soar1-2	0	**a**		*soar1-2*	0	**a**
	soar1-3	0	**a**		*soar1-3*	0	**a**
**0**	OE1	0	**a**	**24**	OE1	0	**a**
	OE3	0	**a**		OE3	0	**a**
	OE6	0	**a**		OE6	0	**a**
	ABI2-OE	0	**a**		ABI2-OE	0	**a**
	Col	0	**d**		Col	0	**e**
	soar1-2	0	**d**		*soar1-2*	0	**e**
	soar1-3	0	**d**		*soar1-3*	0	**e**
**36**	OE1	1.5	**b**	**48**	OE1	17.8	**b**
	OE3	0.8	**c**		OE3	15.6	**c**
	OE6	12.2	**a**		OE6	30.5	**a**
	ABI2-OE	0	**d**		ABI2-OE	5.5	**d**
	Col	13	**e**		Col	25	**d**
	soar1-2	0	**f**		*soar1-2*	3.8	**e**
	soar1-3	0	**f**		*soar1-3*	4.3	**e**
**60**	OE1	78.5	**b**	**72**	OE1	92.6	**a**
	OE3	55. 5	**c**		OE3	76.6	**b**
	OE6	81.7	**a**		OE6	92.7	**a**
	ABI2-OE	36.7	**d**		ABI2-OE	63.3	**c**
	Col	82	**d**		Col	88	**c**
	soar1-2	20.9	**f**		*soar1-2*	22.8	**e**
	soar1-3	38.4	**e**		*soar1-3*	48.5	**d**
**96**	OE1	98.5	**a**	**120**	OE1	99.3	**a**
	OE3	89.8	**c**		OE3	93.8	**b**
	OE6	97.6	**a**		OE6	100	**a**
	ABI2-OE	93.6	**b**		ABI2-OE	98.2	**a**

Table S5. Duncan's multiple range tests for the data of the seed germination rate under NaCl (175 mM) treatment presented in Fig. 2C. Different letters indicate significant differences at $P<0.05$.

Time (h)	Genotype	Mean	Significance of difference 0.05	Time (h)	Genotype	Mean	Significance of difference 0.05
	Col	0	**a**		Col	0	**a**
	soar1-2	0	**a**		*soar1-2*	0	**a**
	soar1-3	0	**a**		*soar1-3*	0	**a**
**0**	OE1	0	**a**	**24**	OE1	0	**a**
	OE3	0	**a**		OE3	0	**a**
	OE6	0	**a**		OE6	0	**a**
	ABI2-OE	0	**a**		ABI2-OE	0	**a**
	Col	0	**c**		Col	0	**e**
	soar1-2	0	**c**		*soar1-2*	0	**e**
	soar1-3	0	**c**		*soar1-3*	0	**e**
**36**	OE1	3.4	**b**	**48**	OE1	24.1	**a**
	OE3	0	**c**		OE3	11.5	**c**
	OE6	9.3	**a**		OE6	21.6	**b**
	ABI2-OE	0	**c**		ABI2-OE	5.4	**d**
	Col	0	**e**		Col	9.8	**d**
	soar1-2	0	**e**		*soar1-2*	1.8	**e**
	soar1-3	0	**e**		*soar1-3*	1.4	**e**
**60**	OE1	82.7	**a**	**72**	OE1	92.2	**a**
	OE3	57.5	**c**		OE3	75.2	**b**
	OE6	78.3	**b**		OE6	92.8	**a**
	ABI2-OE	36.9	**d**		ABI2-OE	70	**c**
	Col	75.7	**e**		Col	84.8	**d**
	soar1-2	25.4	**f**		*soar1-2*	65.4	**e**
	soar1-3	35.9	**f**		*soar1-3*	66.1	**e**
**96**	OE1	99.1	**a**	**120**	OE1	100	**a**
	OE3	88.4	**d**		OE3	92.9	**c**
	OE6	96.9	**b**		OE6	96.9	**b**
	ABI2-OE	91.9	**c**		ABI2-OE	100	**a**

Table S6. Duncan's multiple range tests for the data of the fresh weight presented in Fig. 3C. Different letters indicate significant differences at *P*<0.05.

NaCl (mM)	Genotype	Mean	Significance of difference 0.05	NaCl (mM)	Genotype	Mean	Significance of difference 0.05
**0**	Col	53	**a**	**130**	Col	6.8	**d**
	soar1-2	47	**a**		*soar1-2*	1.17	**e**
	soar1-3	49	**a**		*soar1-3*	1.18	**e**
	OE1	50	**a**		OE1	28.8	**b**
	OE3	53	**a**		OE3	34.4	**a**
	OE6	51	**a**		OE6	27.8	**c**
	ABI2-OE	49	**a**		ABI2-OE	28.7	**b**
	abi1-3/abi2-2	43	**a**		*abi1-3/abi2-2*	0.64	**f**
**150**	Col	4.5	**e**	**175**	Col	1.02	**d**
	soar1-2	0.89	**f**		*soar1-2*	0.33	**f**
	soar1-3	0.87	**f**		*soar1-3*	0.39	**f**
	OE1	23.05	**a**		OE1	15	**b**
	OE3	20	**b**		OE3	16	**a**
	OE6	18	**c**		OE6	13	**c**
	ABI2-OE	16	**d**		ABI2-OE	13.5	**c**
	abi1-3/abi2-2	0.65	**g**		*abi1-3/abi2-2*	0.64	**e**
**200**	Col	0.7	**d**				
	soar1-2	0.18	**e**				
	soar1-3	0.26	**e**				
	OE1	7.9	**a**				
	OE3	8.1	**a**				
	OE6	3.5	**c**				
	ABI2-OE	5.3	**b**				
	abi1-3/abi2-2	0.27	**e**				
